# A pro-inflammatory stem cell niche drives myelofibrosis through a targetable galectin-1 axis

**DOI:** 10.1126/scitranslmed.adj7552

**Published:** 2024-10-09

**Authors:** Rong Li, Michela Colombo, Guanlin Wang, Antonio Rodriguez-Romera, Camelia Benlabiod, Natalie J. Jooss, Jennifer O’Sullivan, Charlotte K. Brierley, Sally-Ann Clark, Juan M. Pérez Sáez, Pedro Aragón Fernández, Erwin M. Schoof, Bo Porse, Yiran Meng, Abdullah O. Khan, Sean Wen, Pengwei Dong, Wenjiang Zhou, Nikolaos Sousos, Lauren Murphy, Matthew Clarke, Aude-Anais Olijnik, Zoë C. Wong, Christina Simoglou Karali, Korsuk Sirinukunwattana, Hosuk Ryou, Ruggiero Norfo, Qian Cheng, Joana Carrelha, Zemin Ren, Supat Thongjuea, Vijay A Rathinam, Anandi Krishnan, Daniel Royston, Gabriel A. Rabinovich, Adam J Mead, Bethan Psaila

**Affiliations:** 1 CAMS Oxford Institute; University of Oxford; Oxford, United Kingdom (UK); 2 Medical Research Council Weatherall Institute of Molecular Medicine (MRC WIMM) and NIHR Biomedical Research Centre Hematology Theme; University of Oxford; Oxford, UK; 3 Human Technopole; Milan, Italy; 4 MRC WIMM Centre for Computational Biology, University of Oxford; Oxford, United Kingdom; 5 Shanghai Key Laboratory of Metabolic Remodeling and Health, Institute of Metabolism and Integrative Biology; Fudan University, Shanghai, China; 6 Qizhi Institute, Shanghai, China; 7 Institute of Cardiovascular Sciences, College of Medical and Dental Sciences; University of Birmingham; Birmingham, UK; 8 Laboratorio de Glicomedicina, Instituto de Biología y Medicina Experimental, Consejo Nacional de Investigaciones Científicas y Técnicas, Buenos Aires, Argentina; 9 Department of Biotechnology and Biomedicine, Technical University of Denmark; Denmark; 10 The Finsen Laboratory, Copenhagen University Hospital; Copenhagen, Denmark; 11 Biotech Research and Innovation Centre, Faculty of Health Sciences, University of Copenhagen; Denmark; 12 Department of Clinical Medicine, University of Copenhagen; Copenhagen, Denmark; 13 Oxford Institute of Biomedical Engineering, Department of Engineering Science, University of Oxford; Oxford, UK; 14 Nuffield Division of Clinical Laboratory Sciences, Radcliffe Department of Medicine, University of Oxford; Oxford, UK; 15 Haematopoietic Stem Cell Laboratory, MRC Weatherall Institute of Molecular Medicine, University of Oxford; Oxford, UK; 16 Department of Immunology, University of Connecticut Health School of Medicine; Farmington, Connecticut USA; 17 Stanford Cancer Institute, Stanford University School of Medicine; Stanford, California, USA; 18 Oxford University Hospitals NHS Trust; Oxford, UK; 19 Facultad de Ciencias Exactas, Físicas y Naturales, Universidad de Buenos Aires, Buenos Aires, Argentina; 20 Ludwig Institute for Cancer Research, University of Oxford, Oxford, UK

## Abstract

Myeloproliferative neoplasms are stem cell-driven cancers associated with a large burden of morbidity and mortality. The majority of patients present with early-stage disease, but a substantial proportion progress to myelofibrosis and/or secondary leukemia, advanced cancers with a poor prognosis and high symptom burden. Currently, it remains difficult to predict progression, and therapies that reliably prevent or reverse fibrosis are lacking. A major bottleneck to the discovery of disease-modifying therapies has been an incomplete understanding of the interplay between perturbed cellular and molecular states. Several cell types have individually been implicated, but a comprehensive analysis of myelofibrotic bone marrow is lacking. We therefore mapped the crosstalk between bone marrow cell types in myelofibrotic bone marrow. We found that inflammation and fibrosis are orchestrated by a ‘quartet’ of immune and stromal cell lineages – with basophils and mast cells creating a TNF signaling hub, communicating with megakaryocytes, mesenchymal stromal cells and pro-inflammatory fibroblasts. We identified the β-galactoside binding protein galectin-1 as a striking biomarker of progression to myelofibrosis and poor survival in multiple patient cohorts, and as a promising therapeutic target, with reduced myeloproliferation and fibrosis *in vitro* and *in vivo* and improved survival following galectin-1 inhibition. In human bone marrow organoids, TNF increased galectin-1 expression, suggesting a feedback loop wherein the pro-inflammatory MPN clone creates a self-reinforcing niche, fueling progression to advanced disease. This study provides a valuable resource for studying hematopoietic cell-niche interactions, with broad relevance for cancer-associated inflammation and disorders of tissue fibrosis.

## Introduction

In most cancers, one or more genetic perturbations are initiating events that confer a survival advantage to the cell-of-origin and its progeny, but the stromal-immune context in which the emergent clone operates determines its ultimate impact. Myeloproliferative neoplasms (MPNs) are initiated by somatic mutations in hematopoietic stem cells (HSCs) that cause clonal expansion and an over-production of blood cells and their progenitors (1). The underlying genetic lesions are well described, with mutations affecting either the gene encoding the Janus kinase signal transducer JAK2 (JAK2V617F), the chaperone protein calreticulin (CALR) or the thrombopoietin receptor (MPL) occurring in almost all patients (2). Interactions between the MPN clone and its microenvironment influence the rate and likelihood of progression to advanced disease (3–5). While most patients present with slow-growing malignancies that only modestly impact life expectancy, some patients develop a severe form of MPN called myelofibrosis. In these patients, fibrotic bone marrow remodeling and pronounced systemic inflammation cause bone marrow failure, extramedullary hematopoiesis, splenomegaly, severe symptoms and a median survival of around 5 years (6).

Myelofibrosis results when cytokines produced by the MPN clone stimulate bone marrow stromal cells to deposit an excess of collagens and other extracellular matrix proteins, consequently destroying the hematopoietic microenvironment. A pivotal role for certain pro-fibrotic and pro-inflammatory growth factors, such as megakaryocyte-derived transforming growth factor β (TGF), is well recognized (7–9). However, the complexity of cell lineages that send and receive the signals that fuel bone marrow inflammation and fibrosis has not been fully elucidated. For example, while various mesenchymal stromal cell (MSC) subsets have been studied, including Nestin+ (10), Gli1+ (11) and Leptin-receptor+ MSCs (12), little is known about the subtypes and transcriptional states of bone marrow fibroblasts in myelofibrosis, and the specific cellular mediators and receptor-ligand (R-L) interactions that lead to pathological stromal cell activation.

Our aim was to build a comprehensive atlas of myelofibrotic bone marrow including hematopoietic stem/progenitor cells (HSPCs), mature hematopoietic cells and their stromal cell neighbors, to identify potentially targetable mediators of inflammation and fibrosis. To achieve this, we first mapped the cellular and molecular cross-talk in myelofibrotic bone marrow at single cell resolution in a mouse model of myelofibrosis, and corroborated findings using bone marrow biopsies and blood samples from patients. Unexpectedly, we found that basophils and mast cells – populations not previously highlighted as important inflammatory drivers in MPNs – are increased in abundance and act as the ‘hub’ for the enhanced TNF signaling (13). We also showed that while MSCs divert to produce extracellular matrix (ECM) components and downregulate their production of hematopoietic support factors, a compensatory increase in production of hematopoietic cytokines occurs from basophils, mast cells and a subset of pro-inflammatory bone marrow fibroblasts (iFibs). Therefore, paracrine hematopoietic support within myelofibrotic bone marrow derives from alternative cellular sources to that in healthy marrow.

The β-galactoside binding protein galectin-1 emerged as one of only two genes differentially expressed in both the MPN clone and the inflamed stroma in myelofibrosis. We confirmed a clear positive correlation between galectin-1 expression and myeloid cancer progression in three large patient cohorts, and showed that inhibition of galectin-1 using a neutralizing anti-galectin-1 monoclonal antibody (mAb) ameliorated myeloproliferation and fibrosis in a mouse model and in 3D, multi-lineage human bone marrow organoids. This identifies galectin-1 as a robust biomarker and a therapeutic target for MPNs and potentially other myeloid malignancies and fibrotic disorders.

## Results

### Generating a high-resolution cellular atlas of myelofibrotic bone marrow

**Figure 1:**
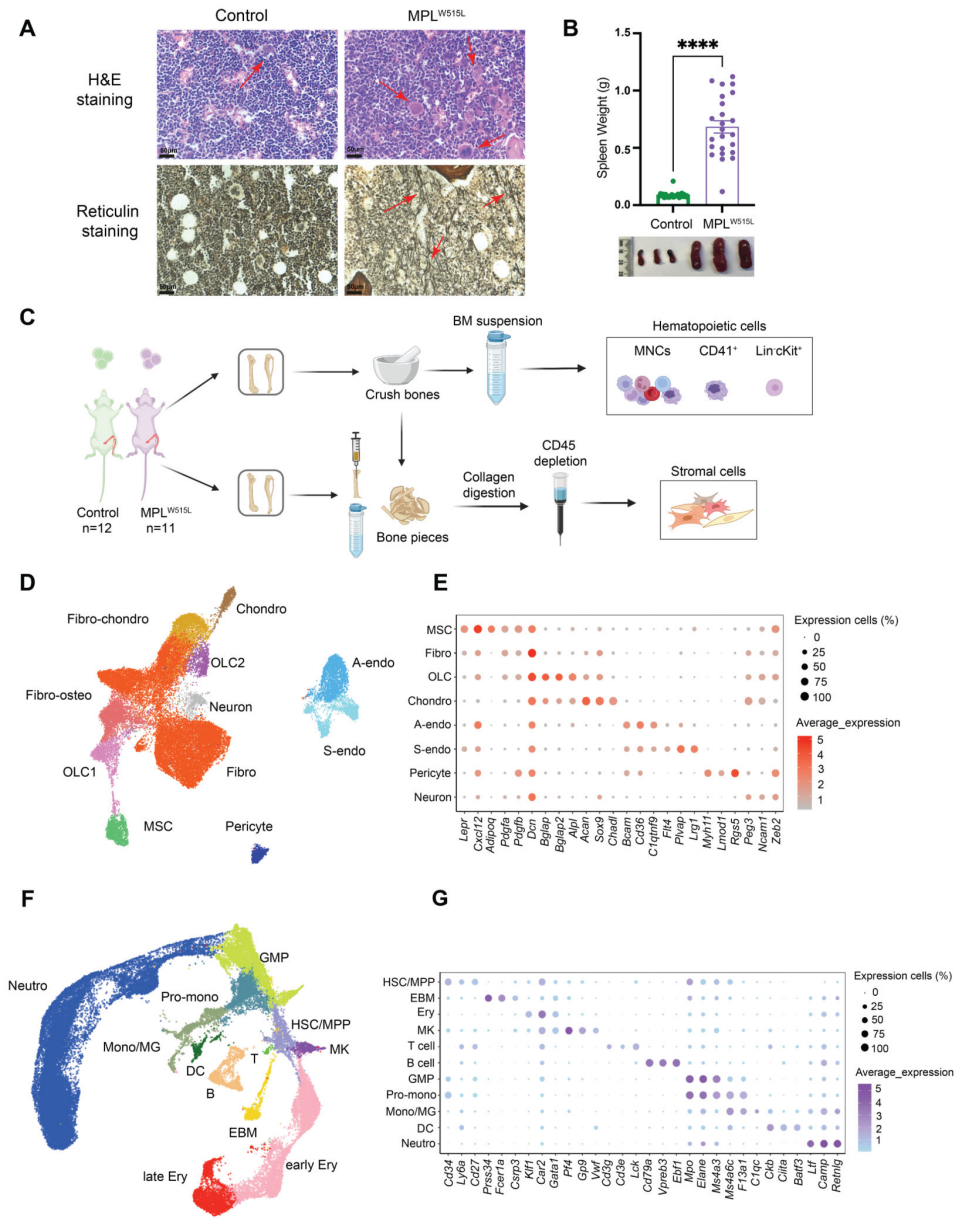
A high resolution cellular atlas of myelofibrotic bone marrow. (**A**) H&E (top) and reticulin stained (bottom) femur sections from control and MPL^W515L^ mice. Red arrows highlight megakaryocytes (top) and reticulin fibrosis (bottom), representative images shown. (**B**) Spleen weights (grams, g) with representative images of control (n = 24) and MPL^W515L^ (n=24) mice. ****p < 0.0001 for unpaired t test with Welch’s correction. Mean ± SEM. (**C**) Schematic of experimental workflow to capture hematopoietic cells including lineage negative (lin-) cKit+ HPSCs, CD41+ and total mononuclear cells, as well as bone marrow stromal cells from control (n=12) and MPL^W515L^ mice (n=11) for single cell RNA sequencing. n=3 independent experiments. Created with Biorender.com. (**D and F**) Uniform Manifold Approximation and Projections (UMAPs) of (**D**) 34,969 stromal cells and (**F**) 42,319 hematopoietic cells from 12 GFP control mice and 11 MPL^W515L^ mice, colored by annotated cell cluster. (**E and G**) Dot plots showing expression of canonical marker genes used to annotate **(E)** stromal and (**G**) hematopoietic cells. Abbreviations: BM, bone marrow; MNC, mononuclear cells; Fibro-chondro, fibroblast-chondrocytes; Chondro, chondrocytes; OLC, osteolineage cells; Fibro-osteo, fibroblast-osteoblasts; Fibro, Fibroblasts; MSC, mesenchymal stromal cells; A-endo, arterial endothelial cells; S-endo, sinusoidal endothelial cells; Neutro, neutrophils; GMP, granulocyte-monocyte progenitors; Pro-mono, monocyte progenitors; Mono/MG, monocyte/macrophages; HSC/MPP, hematopoietic stem and multipotent progenitor cells; MK, megakaryocytes; EBM, eosinophil, basophil, mast cells; DC, dendritic cells; B, B-cell; T, T-cell; Ery, erythrocytes.

To enable detailed analysis of the cellular landscape of myelofibrotic bone marrow, we utilized a well-characterized murine model in which MPL^W515L^, the third most common driver mutation occurring in MPN patients, is introduced into murine HSPCs by retroviral transduction and the cells transplanted into lethally irradiated, wild-type recipients (14). Control mice received HSPCs transduced with GFP alone. As previously described, this resulted in a severe and rapidly progressive myeloproliferative disease that was typically lethal within 4 weeks (14). Mice receiving MPL^W515L^ bone marrow developed leukocytosis, thrombocytosis, polycythemia, pronounced splenomegaly, a reduction in body weight, bone marrow fibrosis, reduced cellularity and increased, atypical megakaryocytes (Figs. 1A and B, S1A). Histology of the spleens revealed loss of the normal lymphoid follicle architecture and markedly increased splenic megakaryocytes and other myeloid cells (Fig. S1B).

We devised a workflow enabling simultaneous capture of hematopoietic and stromal cells from murine femurs, tibiae and iliac crests, and performed high-throughput, droplet-based, single cell RNA-sequencing, isolating total mononuclear cells (MNCs) and enriching for rarer relevant cell types including lineage negative (Lin-) cKit+ HSPCs and cells expressing the megakaryocyte cell surface marker CD41 (Fig. 1C). Capture of non-hematopoietic stromal cells was achieved by performing collagenase digestion of flushed and crushed bone pieces, beads depletion of CD45+ hematopoietic cells and then fluorescence-activated cell sorting (FACS) to isolate the CD45-, Lin-, Ter119-, CD71mid/- non-hematopoietic cell fraction (Fig. 1C, S1C and Table S1) (15).

Following data integration, doublet removal and quality control (Fig. S1D), 77 288 cells were analyzed from 23 mice in 3 independent experiments, including 42 319 hematopoietic and 34 969 stromal cells, generating a comprehensive atlas of normal and myelofibrotic bone marrow (Fig. 1D – G, dataset available via an online data explorer at https://mouse10x.shinyapps.io/myelofibrosis/. Differentially expressed genes for each cluster were calculated after dimensional reduction and clustering, and cell types identified by their expression of canonical marker genes (Fig. 1E and 1G, Fig. S1E, Table S2).

We successfully captured the major cellular subsets annotated in recently published atlases of murine bone marrow (15–18). Within the bone marrow stroma, this included: MSCs (expressing *Lepr, Cxcl12, Adipoq*), fibroblasts (*Dcn, Pdgfra, Pdgfrb*) (19), osteolineage cells (OLC, *Bglap, Bglap2, Alpl*), chondrocytes (*Acan, Sox9*), pericytes (*Myh11, Rgs5*) (20) and neuronal (*Ncam1*) cells, and distinct arteriolar (*Bcam, C1qtnf9*) and sinusoidal (*Plvap, Lrg1*) endothelial cell subtypes (Fig. 1D and 1E, Fig. S1E). Eleven hematopoietic cell types were captured, including hematopoietic stem and multipotent progenitor cells (HSC/MPP, *Cd34, Ly6a, Cd27*), megakaryocytes (*Pf4*), T (*Lck*) and B (*Cd79a, Ebf1, Vpreb3*) lymphocytes, eosinophil/basophil/mast cells (*Prss34, Fcer1a*), erythroid (*Car2, Gata1*), granulocyte-monocyte progenitors and pro-monocytes (*Mpo, Elane*), monocytes/macrophages (*Ms4a6c*) and neutrophils (*Camp, Retnlg*; Fig. 1F & 1G, Fig. S1E).

To compare the cell types captured in our study to previously published datasets of normal (15) and myelofibrotic (21) bone marrow, Symphony analysis (22) was performed, using our data as the reference dataset and projecting cells from existing datasets onto to the reference embeddings. This confirmed annotation in our dataset of several major cell types including fibroblasts, chondrocytes, endothelial, osteolineage, mature neutrophils, eosinophils, basophils, and mast cells that were not captured in previous studies of myelofibrotic bone marrow, particularly in the stromal cell compartment (Fig. S2A and S2B) (18, 21). This dataset therefore represents an unbiased cellular and molecular atlas of the bone marrow in myelofibrosis, enabling a more comprehensive analysis of cellular and molecular interactions and perturbations than has been possible to date.

### Alterations to the cellular constituents of the bone marrow in myelofibrosis

**Figure 2:**
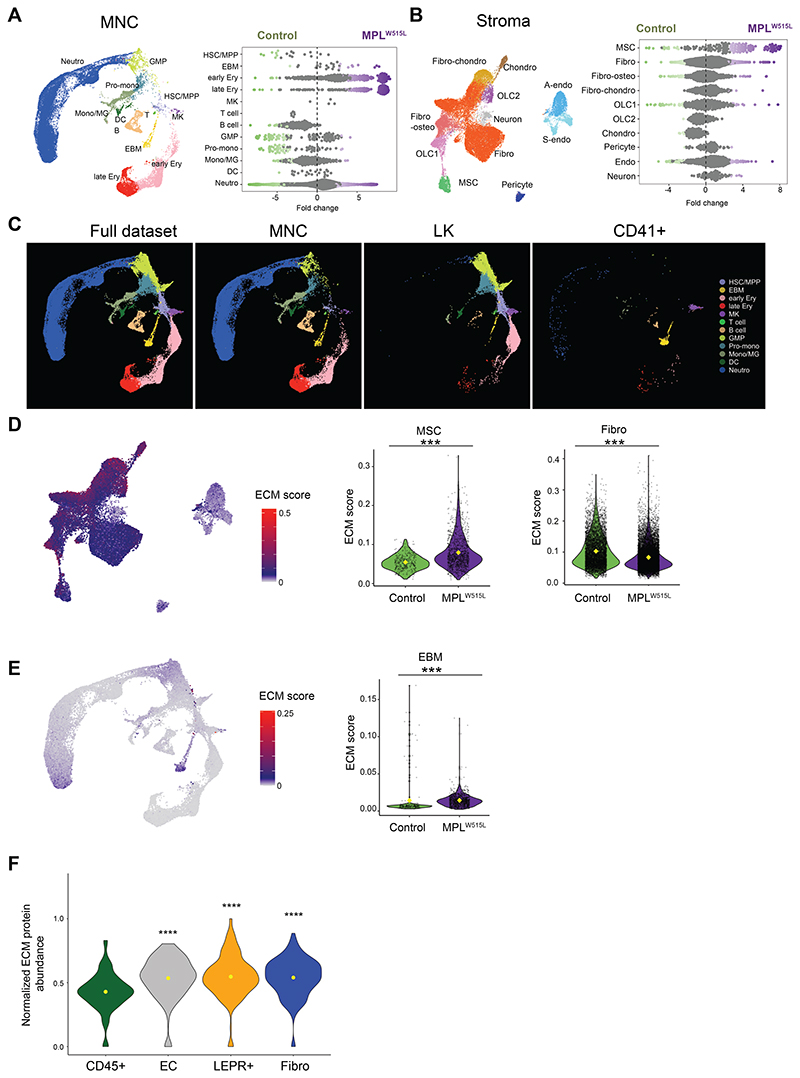
Alterations to the cellular constituents of myelofibrotic bone marrow and source of extracellular matrix components. (**A and B**) Differential abundance of (**A**) mononuclear cell (MNC) subsets and (**B**) stromal cell subsets in control (green) vs. MPL^W515L^ mice (purple), shown with relevant Uniform Manifold Approximation and Projections (UMAPs) to indicate relative frequency of each cell type. Each dot in the differential abundance plots represents a KNN cluster of the indicated cell type, clusters marked green and purple indicate those significantly depleted or enriched in MPL^W515L^ mice respectively. Sinusoidal and arterial endothelial cells are merged (endo) for the purpose of differential abundance in panel B. (**C**) Derivation of the total bone marrow hematopoietic cells captured (full dataset) from the three flow cytometric sorting strategies for MNCs, lineage negative cKit+ HSPCs (LK) and CD41+ cells (CD41), indicating that eosinophil, basophil and mast (EBM) cells and megakaryocytes (MK) were primarily captured by the CD41+ cell sort. (**D and E**) UMAPs (left) showing expression of a gene set of extracellular matrix factors (ECM) in (**D**) stromal and (**E**) full hematopoietic cell dataset, with violin plots (right) showing expression in relevant cell clusters from control (green) and MPL^W515L^ mice (purple). Yellow diamond indicates mean value. ***p < 0.001 for Wilcoxon test. (**F**) Abundance of ECM proteins detected by low-input mass spectrometry proteomics in hematopoietic cells (CD45+), endothelial cells (EC), leptin receptor + mesenchymal stromal cells (LEPR+) and fibroblasts (fibro). **** p < 0001 for adjusted p value comparing each stromal cell subtype to CD45+ hematopoietic cells, n = 4 control mice.

The relative abundance of cell lineages was substantially altered in myelofibrotic bone marrow. In concordance with the expected disease phenotype, erythroid, neutrophil and megakaryocyte cells were substantially expanded in the hematopoietic compartment of MPL^W515L^ mice (Fig. 2A). A pronounced decrease in HSPCs was also observed, with a near absence of B and T lymphocytes (Fig. 2A). Within the stromal compartment, the most striking change in myelofibrosis mice was an expansion of LEPR+ MSCs, with a more modest increase in fibroblasts and decrease in chondrocytes, OLCs and endothelial cells compared to controls (Fig. 2B). We did not find any evidence of monocyte-derived fibrocytes in myelofibrosis bone marrow, with no expression of *Slamf7* or monocyte-affiliated markers detected in stromal cell subsets (Fig. S2C).

Notably, basophils and mast cells were markedly increased in abundance in myelofibrosis mice compared to controls (Fig. 2A). Unexpectedly, we found that the eosinophil, basophil and mast cell (EBM) population had primarily been captured by the enrichment sort for CD41+ cells (Fig. 2C), a canonical cell surface marker of megakaryocyte cells but also expressed on murine basophils at steady-stage, and upregulated after cytokine activation (23).

### Altered cellular sources of ECM components

A defining feature of myelofibrosis is the aberrant deposition of ECM in the bone marrow, causing reticulin fibrosis, bone marrow failure and extramedullary hematopoiesis in particular in the spleen. The specific constituents and cellular origin of ECM factors in normal and myelofibrotic bone marrow have not been well described, although ECM components are recognized as important regulators of HSC function (24). To determine the cellular sources of ECM proteins in the bone marrow, we utilized an ECM gene list derived from proteomic analysis of normal and malignant tissues (25). Higher numbers of ECM genes were expressed by cells from the stroma than the hematopoietic compartment (total ECM genes: n = 233 *vs*. 107; collagens: n = 42 *vs*. 17; glycoproteins: n =159 *vs*. 76 and proteoglycans: n = 32 *vs*. 14 for stroma vs. hematopoietic respectively). Within the stromal cell subsets, high *per cell* expression of ECM genes was detected in all cell types apart from endothelial cells, neurons and pericytes (Fig. 2D, Fig. S2D). Expression of collagen subtypes and glycoproteins was higher in OLCs and chondrocytes than other stromal cell subtypes, while fibroblasts and fibro-chondrocytes were the primary cellular source of proteoglycans, and MSCs predominantly expressed glycoproteins (Fig. S2D).

Expression of ECM components were also detected in the hematopoietic compartment, although in lower abundance than in the stroma (Fig. 2E). Notably, prominent expression of glycoproteins and proteoglycans were detected in EBM cells, as well as a small fraction of monocytes/macrophages and mature neutrophils (Fig. S2D).

To validate these findings at protein level, we performed low-input mass spectrometry proteomics on hematopoietic cells (CD45+) and the key stromal cell subfractions including LEPR+ MSCs, fibroblasts and endothelial cells. 131 ECM proteins were detected in the proteomes, and both the number of ECM proteins detected and their abundance was significantly higher in stromal cell fractions than hematopoietic cells, confirming the stroma as the dominant source of ECM protein in the bone marrow (Fig. 2F). We confirmed that this is also true in human bone marrow by interrogating a recently published atlas capturing both hematopoietic and stromal cell subtypes (26) for expression of ECM proteins (Fig. S2E).

In myelofibrotic bone marrow, *per cell* expression of ECM genes was substantially increased in MSCs and EBM cells but decreased in fibroblasts (Fig. 2D and 2E), suggesting that MSCs and EBM cells are major contributors to the altered deposition of extracellular matrix proteins in myelofibrosis.

### Altered cellular sources of hematopoietic support factors in myelofibrosis

**Figure 3:**
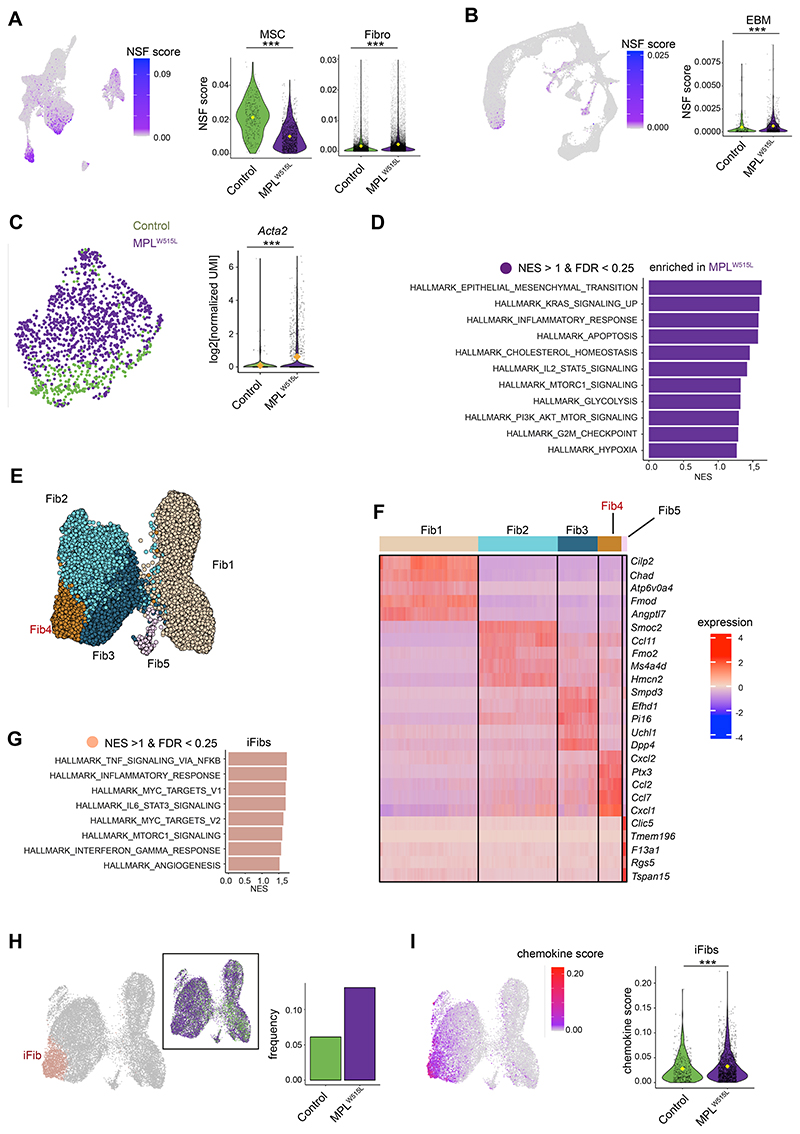
Altered cellular sources of hematopoietic support factors and expansion of inflammatory fibroblasts in myelofibrosis bone marrow. (**A & B**) Uniform manifold Approximation and Projection (UMAP, left) and violin plots (right) showing expression of niche supporting factors (NSFs) in (**A**) stromal and (**B**) hematopoietic cell datasets. Violin plots show expression in mesenchymal stromal cells (MSC), fibroblasts (Fibro) and eosinophil, basophil & mast cells (EBM) from control (green) and MPL^W515L^ mice (purple). ***p < 0.001 for Wilcoxon test. (**C**) MSCs from control (green) and MPL^W515L^ mice (purple) cluster separately, reflecting marked transcriptional reprogramming and myofibroblast trans-differentiation as indicated by increased alpha smooth muscle actin (*Acta2*). (**D**) Significantly enriched HALLMARK gene sets in MSCs from myelofibrosis mice. Selected gene sets shown. (**E**) UMAP showing 5 fibroblast sub-clusters. (**F**) Top 5 differentially expressed genes in each fibroblast subcluster. (**G**) Selected HALLMARK gene sets significantly enriched in Cluster 4, reflecting inflammatory fibroblast (iFib) phenotype. (**H**) Frequency of iFibs in MPL^W515L^ *vs*. control mice. (**I**) Expression of chemokine genes in fibroblasts. ***p < 0.001 for Wilcoxon test.

Bone marrow Lepr+ MSCs transdifferentiate into myofibroblasts in myelofibrosis in response to platelet derived growth factor receptor (PDGFR) stimulation, downregulating their production of hematopoietic niche support factors in parallel with their increased expression of fibrogenic and osteogenic genes (12, 21). We detected clear transcriptional reprogramming of MSCs in myelofibrotic bone marrow, with a pronounced reduction in expression of hematopoietic niche support factors (Fig. 3A, Table S4) in parallel with the increased expression of ECM factors (Fig. 2D). While the reduction in expression of hematopoietic support factors by MSCs in myelofibrosis has been documented (12, 21, 27), prior studies did not examine whether the production of hematopoietic support factors ‘shifts’ from MSCs to other cellular components of the bone marrow niche. We found that the reduction in gene expression of niche support factors (NSF) from MSCs was compensated by a significant increase in expression of NSF in fibroblasts and also EBM cells in myelofibrosis *vs*. control cells (Figs. 3A and 3B). The *per cell* expression of NSF, in particular *Cxcl12* and *Csf1*, was markedly decreased in myelofibrosis vs. control bone marrow MSCs but increased in fibroblasts (Figs. S3A and S3B) at transcript level.

Myelofibrosis MSCs were transcriptionally distinct, with significant enrichment of genes and pathways associated with myofibroblast transition including *Acta2*, KRAS and phosphoinositide-3-kinase (PI3K) signaling, inflammatory response genes and IL2-STAT5 signaling (Figs. 3C and 3D). Therefore, myelofibrosis-induced MSC trans-differentiation leads to increased ECM production but reduced hematopoietic support from MSCs. Assuming that protein level expression reflects the observed changes in gene expression, this suggests that hematopoiesis is guided by alternative cellular sources in the setting of MPNs, potentially influencing the competitive advantage of the MPN clone over healthy hematopoiesis.

### Emergence of a distinct inflammatory fibroblast subset in the myelofibrotic niche

The relative proportion of fibroblast cells overall was only minimally increased in myelofibrotic bone marrow (Fig. 2B). As fibroblasts were the most abundant stromal cell type captured, and as distinct fibroblasts subsets have been reported to be important in other pathologies (28, 29), we selected the fibroblasts for further analysis, confirming their expression of the canonical fibroblast markers *Pdgfra/Pdgfrb* and performing unsupervised sub-clustering (Fig. S3C).

Five transcriptionally distinct sub-clusters were identified (Fig. 3E, Table S2), of which one cluster (Fib4) uniquely showed striking enrichment for inflammatory pathways (Fig. 3F and 3G) and was therefore annotated as representing inflammatory fibroblasts (iFibs). iFibs were significantly enriched for TNF signaling via NFKB, inflammatory response signaling, IL6-JAK-STAT3 signaling and interferon gamma response (Fig. 3G). Pseudotime analysis showed that iFibs arise via a separate trajectory from Fib1 cluster (Fig S3D). The relative frequency of iFibs was 2-fold higher in myelofibrosis mice than controls (Fig. 3H), and expression of chemokine genes was strongly enriched in the iFibs with significantly increased per cell expression of chemokines in MPL vs. control fibroblasts (Fig. 3I, Table S3), including *Kitl, Cxcl12, Ccl2, Cxcl1* (Figs. S3A and S3E). The iFib cluster also expresses *Cxcl5*, which has been identified in a recent fibroblast atlas as a marker for perturbation-specific, activated fibroblast states and not detected in steady-state fibroblasts (19) (Fig. S3F). Collectively these data support that, although overall fibroblast numbers are only slightly altered in myelofibrosis, distinct inflammatory fibroblast subsets producing hematopoietic support factors are markedly expanded in number, thereby contributing to the development of an aberrant hematopoietic niche in myelofibrosis.

### Expanded pro-inflammatory basophils, mast cells and megakaryocytes in myelofibrosis

**Figure 4:**
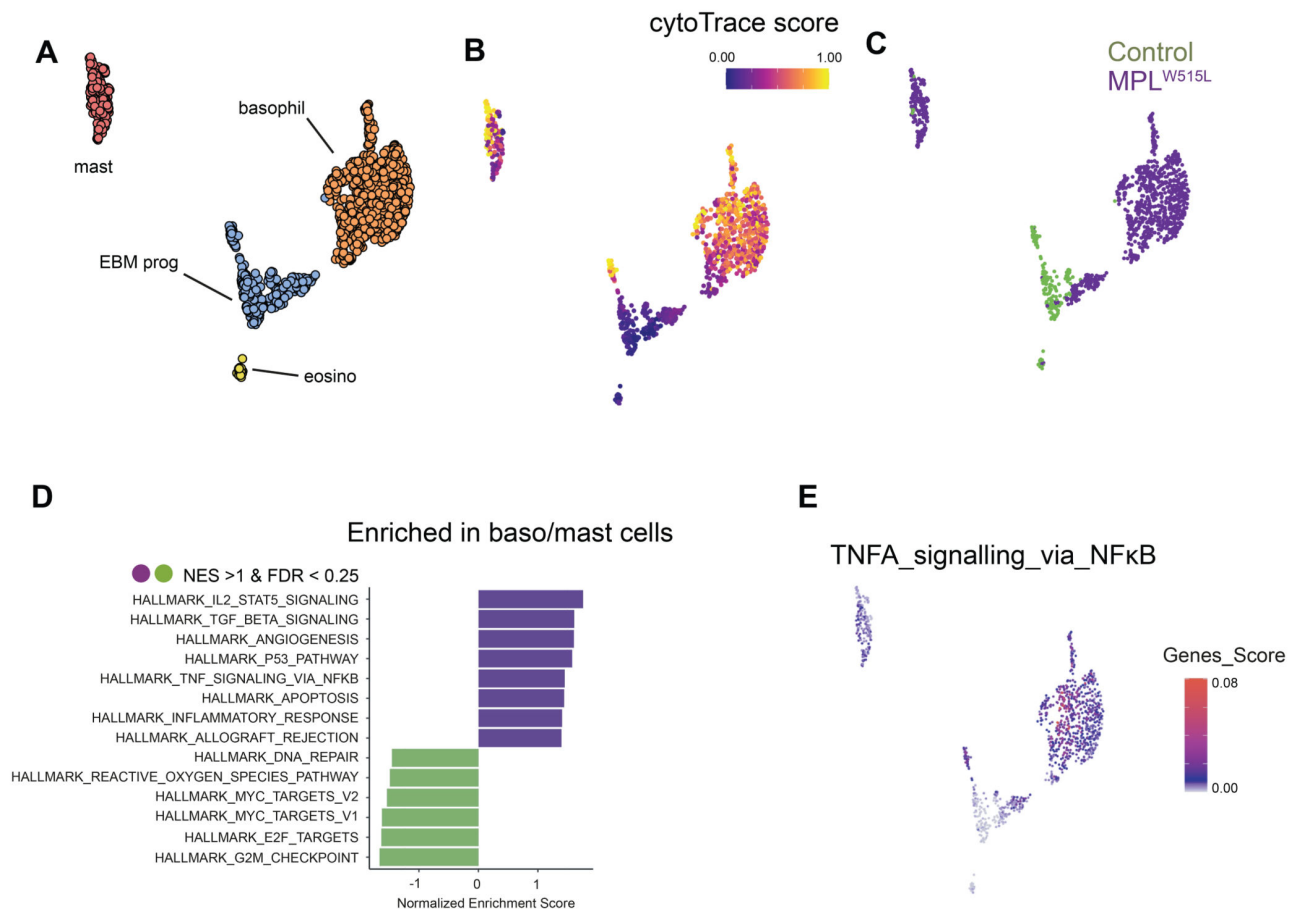
Expansion of pro-inflammatory basophils and mast cells in myelofibrosis. **(A)** UMAP showing annotated sub-clusters of cells from the eosinophil, basophil and mast (EBM) cell cluster. (**B**) CytoTRACE differentiation state analysis of EBM cells, with blue indicating primitive state and yellow showing differentiation trajectory. (**C**) UMAP identifying cells originating from MPL^W515L^ (purple) and control (green) mice. (**D**) Significantly enriched HALLMARK gene sets in basophils and mast cells from MPL^W515L^ vs. control mice. (**E**) Expression of TNF-NFκB pathway genes projected onto the EBM cell UMAP.

Megakaryocyte proliferation and morphological atypia are hallmark features of overt and pre-fibrotic myelofibrosis (1), and we found an expansion of megakaryocytes with angiogenic, proliferative and inflammatory gene expression programs in the myelofibrosis mice (MK3, 4 and 5, Fig. S4A – E). While megakaryocytes are well recognized as important drivers of fibrosis (9, 30), the pathological contributions of basophil and mast cell subsets in myelofibrosis have not been extensively studied (31). Having noted a significant increase in the abundance of EBM cells (Fig. 2A), we extracted cells from the EBM cluster for a more detailed analysis (Fig. 4A). Four distinct subtypes of EBM cells were annotated – EBM progenitors, mast cells, basophils and a small population of mature eosinophils (Figs. 4A and 4B, and Fig. S4F, Table S2). The relative proportions and transcriptional activity of these cellular subsets were distinct in myelofibrosis bone marrow, with a dramatic expansion of basophils and mast cells, and relatively few eosinophils in myelofibrosis mice compared to controls (Fig. 4C), and significant enrichment of IL2-STAT5, TGFB, and TNF via NF-κB inflammatory signaling pathways (Figs. 4D and 4E).

### Basophils and mast cells emerge as the ‘hub’ of TNF and pro-inflammatory cytokine signaling

**Figure 5:**
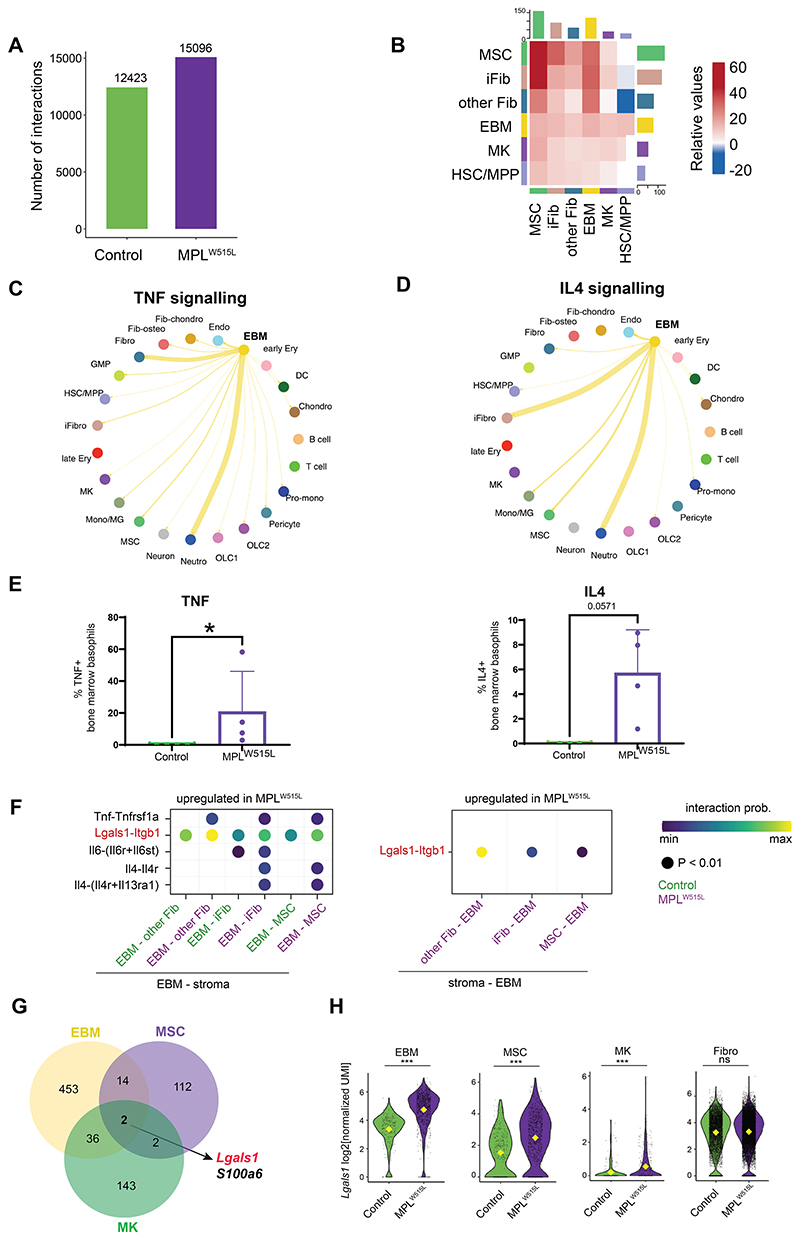
Basophils and mast cells emerge as the ‘hub’ of TNF and interleukin 4 pro-inflammatory signaling. (**A**) Number of inferred Ligand (L) – Receptor (R) interactions in control and MPL^W515L^ bone marrow. (**B**) Differential number of L-R interactions in MPL^W515L^ vs. control bone marrow. Total Number of enriched L-R interactions is shown as a bar on the x/y axes and relative strength of the interactions (MPL^W515L^ vs. control bone marrow) is shown in the heatmap for key stromal and hematopoietic cell populations. (**C & D**) Circus plot depicting interaction pathway of (**C**) TNF and (**D**) IL4 uniquely upregulated in MPL^W515L^ mice. The width of the connections reflects the strength of the interactions between two populations. (**E**) Percentage of TNF-positive (left) and IL4-positive (right) basophils in MPL^W515L^ vs. control bone marrow (n=4) assessed by intracellular flow cytometry. * p < 0.05. (**F**) Selected L-R interactions predicted to be upregulated in MPL^W515L^ mice between EBM cells, fibroblasts, iFibs, and mesenchymal stromal cells (MSCs). **(G)** Venn diagram showing distinct and overlapping differentially expressed genes in EBM, MSC and MK clusters. (**H**) Violin plots showing expression of *Lgals1* in EBM, MSC, MK and fibroblasts in control and MPL^W515L^ mice. Abbreviations: R – L, receptor-ligand; L – R, ligand-receptor; TNF, tumor necrosis factor alpha; IL, interleukin; EBM, eosinophil, basophil, mast cells; iFibs, inflammatory fibroblasts; MSCs, mesenchymal stromal cells; Fibro, fibroblast; HSC/MPP, hematopoietic stem and multipotent progenitor cells. ***p < 0.001; ns – non-significant for Wilcoxon test.

To identify how the cellular cross-talk was altered in myelofibrotic bone marrow, we computationally inferred the interacting receptor-ligand (R-L) pairs that might mediate communication between cell types (32). The overall number of predicted R-L interactions was 20% higher in MPL^W515L^ than control mice (Fig. 5A), and the aberrant signaling was largely due to increased interactions deriving from basophils, mast cells and megakaryocytes in the hematopoietic compartment and MSCs and inflammatory fibroblasts in the stroma (Fig. 5B), highlighting these 4 cell types as ‘orchestrators’ of inflammatory signaling in myelofibrotic bone marrow. Basophils and mast cells emerged as the hub of TNF and IL4 signaling in MPL^W515L^ mice, with fibroblasts, inflammatory fibroblasts, MSCs and neutrophils and monocytes/macrophages as their key interacting partners (Figs. 5C and 5D, Fig. S5A & S5B). Intracellular flow cytometry confirmed increased TNF and IL4 protein abundance in MPL^W515L^ basophils compared to controls (Fig. 5E & S5C). This effect was seen both in basophils analysed *ex vivo* from bone marrow cells of MPL^W515L^ mice (Fig. 5E) as well as basophils differentiated *ex vivo* from MPL^W515L^ vs. control HSPCs (Fig. S5C), indicating that the induction of TNF and IL4 occurred as a direct effect of the MPN driver mutation and was not dependent on niche reprogramming.

*Lgals1*, the gene encoding the protein galectin-1, a β-galactoside binding protein which interacts with β-1 integrin (*Itgb1*), emerged as a R-L pair with substantially enhanced predicted signaling across the key interacting cell types (Fig. 5F and Figs. S5D and S5E). When we looked for genes which were differentially expressed in the key interacting cell types in myelofibrosis, only two genes were concordantly dysregulated across cell types – *S100a6* and *Lgals1* (Fig. 5G). A role for *S100a6* and other *S100* family members in inflammation and malignant hematopoiesis has previously been reported (33–35), whereas galectin-1 has not been extensively studied in myeloid malignancies. Expression of *Lgals1* was strikingly increased in basophils and mast cells, MSCs and megakaryocytes in MPL^W515L^ mice compared to control mice, with high levels of expression in fibroblasts overall but no significant difference in per cell expression level (Fig. 5H). *Lgals1* was also highly expressed in monocytes, pro-monocytes and GMPs, but neither the abundance of these cell types nor the *per* cell *Lgals1* expression was increased in myelofibrosis, indicating that monocytes and their precursors are not a source of excess galectin-1 production in myelofibrosis (Fig. S5F). Together, these data suggested that galectin-1 signaling might play a key pathological role in myelofibrosis progression and warranted further exploration.

### Galectin-1 inhibition ameliorates myelofibrosis disease phenotype *in vivo*

**Figure 6:**
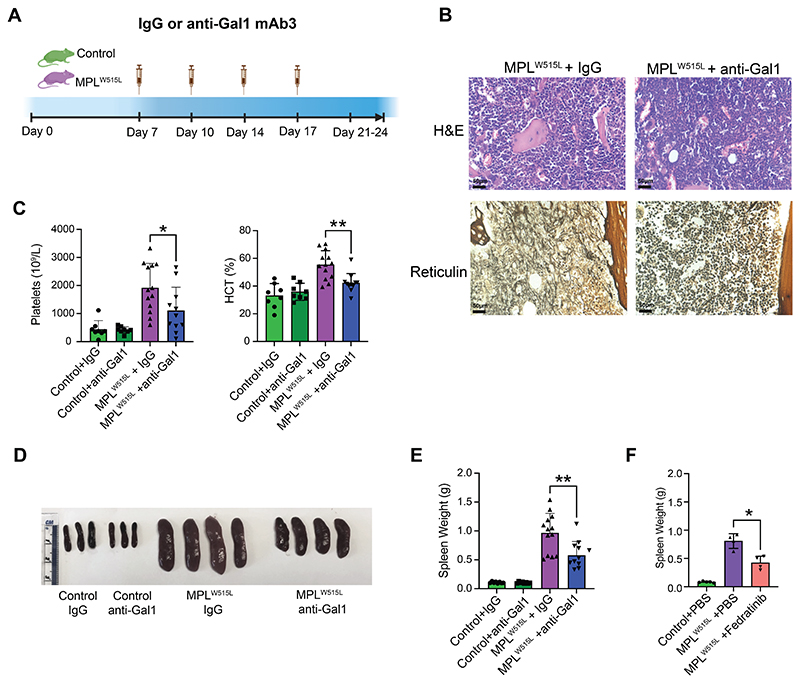
Inhibition of Galectin-1 ameliorates fibrosis and myeloproliferation *in vivo* (**A**) Schematic of treatment with isotype control (IgG) or anti-galectin-1 (anti-Gal1) mAb3, initiated on day 7 following transplantation of control or MPL^W515L^ BM cells. (**B**) H&E and reticulin staining of femur sections from MPL^W515L^ mice treated with IgG control or anti-Gal1 mAb3. Representative images shown. (**C**) Mean ± SEM platelet counts and hematocrit (HCT) in IgG or anti-Gal1 treated control (n=8 and n=8) and MPL^W515L^ mice (n=13 and n=11). *p < 0.05, **p < 0.01 for unpaired t test with Welch’s correction.. (**D & E**) Representative images (**D**) and weights (**E**) of spleens from IgG or anti-Gal1 mAb3 treated control (n=8 and n=8) and MPL^W515L^ mice (n=13 and n=11). (**F**) Mean + SEM spleen weights of mice treated with PBS control (n=5) or the JAK2 inhibitor fedratinib (n=4). *p < 0.05, **p < 0.01 for unpaired t test with Welch’s correction.

To test whether galectin-1 signaling contributes to the pathobiology of myelofibrosis *in vivo*, we tested the impact of a neutralizing anti-galectin-1 monoclonal antibody (Gal-1-mAb3) that binds to a specific sequence in galectin-1 not present in other galectin family proteins (36) in the MPL^W515L^ mouse model. Control and MPL^W515L^ mice were treated with either IgG isotype control or Gal-1-mAb3 by intraperitoneal injection (Fig. 6A). Galectin-1 neutralization led to a reduction in bone marrow fibrosis and cellular architecture in the MPL^W515L^ mice (Fig. 6B), and reduced the myeloproliferative phenotype with significantly reduced thrombocytosis, polycythemia and splenomegaly (Fig. 6C – E). Notably, the reduction in splenomegaly with Gal-1-mAb3 treatment was similar to that with fedratinib, a JAK2 inhibitor in clinical use (Fig. 6F), and no cytopenias were observed following galectin-1 inhibition in the control mice (Fig. 6C), indicating specific inhibition of the MPN clone rather than a non-specific cytoreductive impact. Furthermore, inhibition of galectin-1 led to significantly improved MPN-free survival (Fig. S6A).

### Galectin-1 is a robust biomarker of fibrosis progression in patients with MPNs

**Figure 7:**
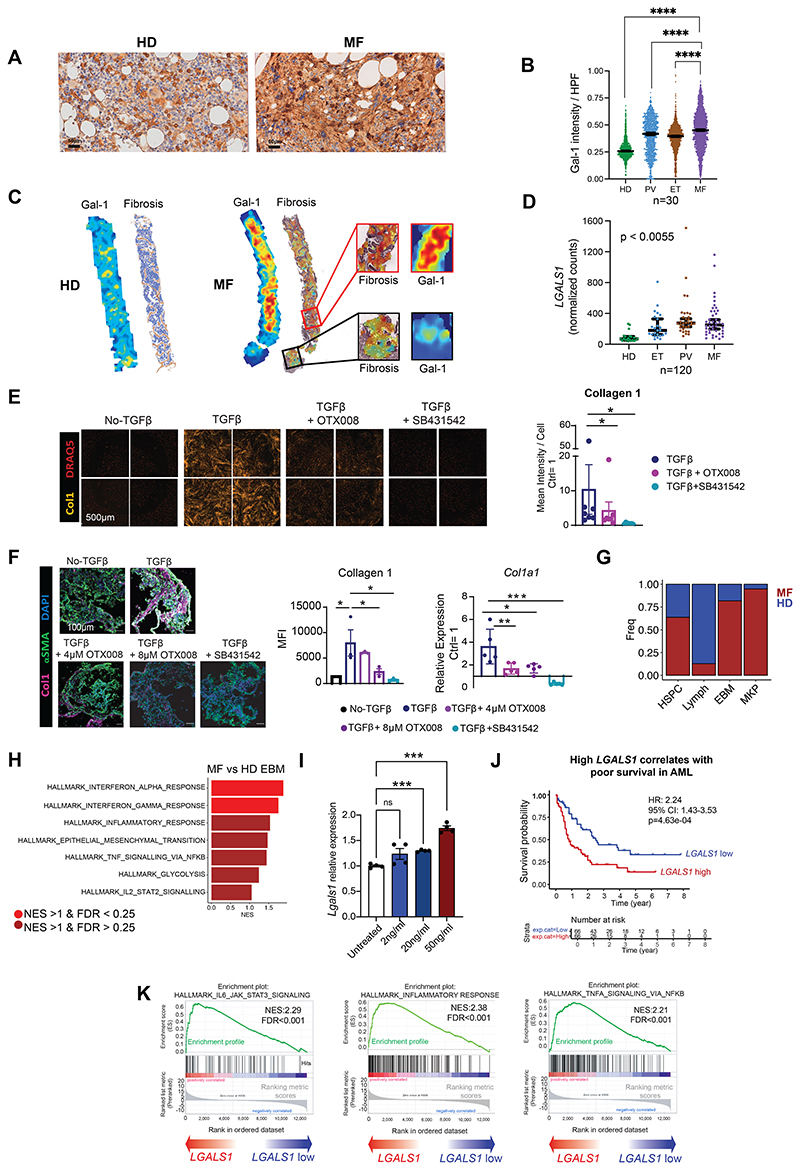
Galectin-1 is a robust biomarker for fibrosis and poor outcomes in myeloid malignancies. **(A)** Representative immunohistochemistry staining for galectin-1 (Gal-1) in healthy donor (HD) (n=7) and myelofibrosis (MF) (n=14) bone marrow biopsy sections. **(B)** Gal-1 expression per high power field (HPF) view in bone marrow biopsy sections from HDs (n=7) and patients with essential thrombocythemia (ET, n = 9), polycythemia vera (PV, n=7), and MF (n=14). Median + 95% CI shown, ****p < 0.0001 for Kruskal-Wallis test. **(C)** Gal-1 staining intensity correlated with reticulin fibrosis density across bone marrow biopsy sections from HDs and MF patients. Color scale from blue to red as fibrosis density increases. Representative images shown. **(D)** LGALS1 expression in platelets from a cohort of 120 HDs and patients with MPNs (HD=21, ET=24, PV=33, MF=42). Median + 95% CI. (**E**) TGFβ-induced fibroblast to myofibroblast transition assay using human BMSCs treated with TGFβ alone + OTX001 (galectin-1 inhibitor) or SB431542 (TGFβ inhibitor). Representative images shown for high-throughput, 384-well imaging plate (left). Each treatment was performed in quadruplicate and 4 images acquired per well (n=7 patients). Chart (right) shows MFI per cell for collagen 1 normalized to the no-TGFβ control ± SEM (n=7). *p < 0.05 for wilcoxon matched pairs signed rank test. (**F**) Impact of OTX008 on TGFβ-induced Collagen 1 and aSMA in human iPSC-derived BM organoids. Representative images (left); Mean + SEM for protein/mRNA expression quantification of Collagen 1/*COL1A1* (right). n=5-8 organoids from 3 independent experiments. *p < 0.05, **p < 0.01, ****p < 0.0001 for one-way ANOVA. (**G**) Bar chart showing relative proportion of cell subtypes from a previously published dataset of ~120,000 cells from human CD34+ hematopoietic stem/progenitor cells from patients with myelofibrosis (MF) and age-matched healthy donors (HD). (**H**) Enriched HALLMARK gene sets in EBM progenitor cells from MF patients *vs*. HD. Abbreviations: UMAP, Uniform manifold Approximation and Projection; EBM, eosinophil (eosino)-basophil (baso)-mast cells; MF, myelofibrosis; HD, healthy donors; NES, normalized enrichment score; FDR, false discovery rate. **(I)** Mean ± SEM *Lgals1* mRNA expression in human bone marrow organoids with/without treatment with TNF at doses shown. n=80 organoids across 2 independent experiments. *p < 0.05, **p < 0.01, ****p < 0.0001 for one-way ANOVA. (**J**) Schematic illustrating the interactions between basophils, mast cells and megakaryocytes derived from the MPN clone interacting with BMSC subsets, fueling inflammation and fibrosis via galectin-1 induction. Created with Biorender.com. Abbreviations: Ctrl, control; TGFβ, transforming growth factor β; Col1, collagen 1; αSMA, alpha smooth muscle actin; anti-Gal-1, monoclonal anti-Galectin-1 neutralizing antibody; HCT, hematocrit; IgG, isotype IgG control antibody; H&E, hematoxylin and eosin; g, grams. (**K**) Kaplan-Meier survival curves showing correlation between high *LGALS1* expression and poor survival in 132 patients with acute myeloid leukemia (AML) in The Cancer Genome Atlas (TCGA) dataset. (**L**) Gene set enrichment analysis show significant enrichment of IL6-JAK-STAT3 signalling, inflammatory response and TNF signalling via NFKB in patients with high *LGALS1* expression in TCGA database. Abbreviations: HR, hazard ratio; NES, normalized enrichment score; FDR, false discovery rate.

Given the amelioration of disease phenotype *in vivo* in the mouse model, we next sought to validate galectin-1 in myeloid malignancies in the setting of human disease, using a series of patient cohorts (Table S4). We first tested whether galectin-1 expression correlated with fibrosis progression in patients with myeloproliferative neoplasms, quantifying galectin-1 protein in bone marrow biopsies of 30 patients, including those with myelofibrosis (n = 14), non-fibrotic MPNs (essential thrombocythemia [ET], n = 9 and polycythemia vera [PV], n = 7) and age-matched healthy controls (n = 7, Table S4). Galectin-1 was markedly increased in myelofibrotic bone marrow (Fig. 7A). Objective quantification of staining intensity per high power field view showed a significant increase in galectin-1 with progression to myelofibrosis across patient groups (Fig. 7B, P < 0.001 for myelofibrosis vs. healthy donors and P < 0.001 for myelofibrosis vs. ET and PV). Bone marrow fibrosis is often unevenly distributed in the bone marrow space, and this heterogeneity is inadequately captured by the standard categorical fibrosis grading system that is typically employed in clinical assessments (e.g. WHO grade MF 0 – 3). In order to measure the association between galectin-1 expression and reticulin fibrosis more precisely, we employed a recently developed machine learning pipeline that enables automated fibrosis quantification by allocating a Continuous Index of Fibrosis (CIF) score for each bone marrow region, creating a heatmap representing the density of fibrosis across the entire marrow specimen (37). This showed clear correlation between the intensity of galectin-1 immunostaining and the density of fibrosis within the marrow sections (Fig. 7C), as well as between patient samples (Fig. 7B).

To further validate galectin-1 as a biomarker and to see if it could be utilized as a non-invasive peripheral blood biomarker of fibrosis, we investigated galectin-1 expression in a large cohort of 120 patients where platelet transcriptomes were available from patients with myelofibrosis (n=42), ET (n=24), PV (n=33) and healthy controls (n=21, Table S4) (38). A progressive and highly significant increase in galectin-1 expression was observed with progression of MPN to fibrosis (monotonic trend from controls to PV/ET to myelofibrosis, P < 0.0055, Fig. 7D), with a 3.4-fold increase in myelofibrosis vs. controls.

### Galectin-1 validates as a targetable mediator of fibrosis in human cellular assays and bone marrow organoids

MPN mouse models are useful surrogates for the human disease, but evidence that a potential target can be functionally validated using human experimental systems is more compelling. We therefore explored whether galectin-1 was mediating a severe disease phenotype using human disease models. We derived bone marrow stromal cells (BMSCs) from marrow aspirates of patients with MPNs (Table S4) and utilized these in a TGFβ-induced fibroblast-to-myofibroblast transition assay (39). Treatment of BMSCs with recombinant human TGFβ led to increased collagen 1 deposition and αSMA expression, which was reversible on inhibition of TGFβ signaling with SB431542, an inhibitor of the TGFβ activin receptor-like kinase (ALK) receptors (40) (Fig. 7E). OTX008, a small molecule galectin-1 inhibitor previously shown to inhibit pulmonary fibrosis, inhibited TGFβ-induced fibroblast-to-myofibroblast transition (Fig. 7E and Fig. S6B) (41).

To confirm a role for galectin-1 as a mediator of TGFβ-induced bone marrow fibrosis in a multi-cellular bone marrow microenvironment, we utilized a three dimensional model that better recapitulates the complexity of human bone marrow. Bone marrow organoids were generated from human induced pluripotent stem cells using an optimized protocol that gives rise to the key stromal and hematopoietic cellular elements of the central marrow space, approximating the transcriptional and architectural features of the native human hematopoietic tissues (42). In this model, OTX008 significantly inhibited TGFβ-induced collagen 1 and αSMA expression at both protein and mRNA level (Fig. 7F and Fig. S6C).

### TNF upregulates galectin-1 gene expression

Given the pronounced increase in TNF signaling from basophils and mast cells in the MPL^W515L^ mouse model (Figs. 4E and 5C), and as TNF-NF-κβ signaling has previously been shown to regulate LGALS1 expression by T cells (43), we hypothesized that TNF might stimulate galectin-1 production in human bone marrow. We first corroborated that basophils and mast cells were increased in frequency and had an inflammatory phenotype in the setting of myelofibrosis in patients by interrogating a scRNAseq dataset of ~120,000 CD34+ Lin- HSPCs isolated from a cohort of 15 myelofibrosis patients and 6 age-matched healthy donors (9). A population of EBM progenitors was identified (Fig. S6D, Table S3), which were significantly more abundant in myelofibrosis patients than healthy controls (Fig. 7G). Similar to our findings in the mouse model, these cells showed a striking enrichment of inflammatory response, IL2-STAT and TNF signaling (Fig. 7H). Notably, a significant increase in *LGALS1* was observed in patients with myelofibrosis due to either *JAK2V617F* or *mutCALR* (Fig. S6E), confirming that basophils and mast cells are likely to play an important role in the pathobiology of myelofibrosis in patients and contribute to TNF pro-inflammatory pathways.

TNF is a potent activator of nuclear factor (NF)-κB (44), and NF-κB directly binds to regulatory elements in exon 1 of the *LGALS1* gene, enhancing gene expression (43). We therefore tested whether the mechanism of galectin-1 increase in myelofibrosis might occur secondary to TNF stimulation. Indeed, TNF treatment of bone marrow organoids robustly led to a dose-dependent increase in *LGALS1* expression (Fig. 7I), suggesting a model wherein a self-reinforcing, inflammatory MPN niche is created by expanded populations of basophils, mast cells, MSCs and inflammatory fibroblasts with a central role for TGFβ, TNF and galectin-1 signaling (Fig. 7J).

### High galectin-1 is associated with poor survival in *de novo* acute myeloid leukemia and progression to blast phase MPN

Given the disease modifying activity of the anti-galectin-1 antibody treatment in the MPL^W515L^ mouse model, we hypothesized that high expression of galectin-1 may be detrimental more broadly in myeloid malignancies. We therefore interrogated The Cancer Genome Atlas (TCGA) to test whether expression of galectin-1 correlated with overall survival in 132 patients with acute myeloid leukemia (45). There was a clear correlation between *LGALS1* expression level and poor survival (Fig. 7K, P < 0.0005), with highly significant enrichment of inflammatory signaling pathways in patients with high *LGALS1* levels and poor survival, including inflammatory response, IL6 – JAK – STAT signaling and TNF signaling (Fig. 7L). We also found significantly increased *LGALS1* expression in accelerated/blast phase MPN (AP/BP-MPN), suggesting a role for galectin-1 in leukemic progression of MPN. *LGALS1* expression was significantly increased in RNA-sequencing data from 200-cell ‘mini-bulks’ of CD34+ HSPCs from patients with AP/BP-MPN (n=10, pre-treatment) vs healthy controls (n=5, Log2FC 2.09; p=0.01, Suppl. Fig. S6F) (46). Interrogating a scRNAseq dataset capturing both HSPCs and total MNCs from healthy donors (13,713 cells) and patients with AP/BP-MPN (44,107 cells) revealed significantly increased *LGALS1* expression in HSCs, multipotent progenitors and megakaryocyte-erythroid progenitors but not granulocyte-monocyte progenitors or mature blood cell lineages (Suppl. Fig. S6G).

Collectively, these results highlight galectin-1 as a central pathological mediator in myeloid malignancies, a promising biomarker, and a therapeutic target that may alter the disease course, which is not possible to achieve for the majority of patients using currently available medical therapies.

## Discussion

MPNs are inflammatory pathologies that result in a significant burden of morbidity and mortality. The majority of patients present with early-stage malignancies, presenting an opportunity for intervention. However, at present, there are no drug therapies that robustly impede or reverse progression to fibrosis, and a more detailed understanding of the genetic and non-genetic drivers of MPN progression is crucial. In this study, we present a comprehensive road-map of the cellular composition of myelofibrotic bone marrow, providing a platform for the discovery and characterization of novel cellular and molecular targets for therapy. Although prior studies highlighted important aspects of disease pathophysiology (9, 18, 21), these datasets have not simultaneously captured hematopoietic and stromal cells, precluding accurate delineation of the multi-lineage interactions that occur between myeloid cells of the MPN clone and components of their niche. The analyses presented here revealed perturbations to cellular frequencies and transcriptional phenotypes that were previously unappreciated, noting that an expansion of basophils, mast cells and a distinct subset of inflammatory fibroblasts collectively underlie pathogenic cellular interactions in myelofibrosis.

In individuals who acquire an MPN cancer driver mutation, the inflammatory microenvironment is an important determinant of clinical phenotype, symptom severity and the risk of disease progression (47). The same mutations can present with diverse clinical phenotypes, including in healthy individuals without overt hematologic disease (48). Although specific genetic contexts (high molecular risk mutations e.g., concurrent *ASXL1, SRSF2* mutations or a high *JAK2V617F* allele burden) increase the likelihood of progression to fibrosis, these are not essential, suggesting a major role for cell-extrinsic signaling in driving disease evolution. Recent studies revealed that MPN driver mutations are typically acquired early in life, often several decades before clinical presentation (49–51), yet myelofibrosis usually presents in the later decades of life. One explanation for the long latency observed between mutation acquisition and clinically overt disease is that the composition and function of the bone marrow stroma becomes more permissive for MPN outgrowth with age. A pro-inflammatory, TGFβ-rich stroma (52) and reduced MSC-derived hematopoietic support factors (53) develop with physiological ageing and induce a myeloid bias even in individuals without an MPN driver mutation. Here, we show that an MPN induces an exacerbation of the inflammatory and myeloid-biased hematopoiesis phenotype that occurs as part of healthy ageing (54), encouraging speculation that aging might accelerate the development of the self-reinforcing, malignant niche in myelofibrosis (55).

We demonstrate the utility of the dataset in identifying clinically-actionable targets by focusing on galectin-1, a β-galactoside binding protein that has been previously implicated in cancer, tissue fibrosis and immunoregulation (56, 57) although its role in myeloid malignancies has not been fully investigated. Exploration of galectin-1 expression levels in large patient cohorts showed a clear association with fibrosis progression and significant correlation with survival in patients with myeloid leukemias. A functional role for galectin-1 was confirmed, using 2D and 3D *in vitro* models of bone marrow fibrosis and also *in vivo* by demonstrating efficacy of a neutralizing anti-galectin-1 mAb (36).

Previous studies have suggested modes of action for galectin-1 that may be relevant in myeloid malignancies. Galectin-1 has been identified as a mediator of TGFβ- and hypoxia-induced lung fibrosis (41), and direct anti-proliferative effects have been shown using shRNA knock-down of galectin-1 as well as treatment with OTX008, a small molecule inhibitor that reached phase I clinical trials for patients with advanced solid tumors. Proliferative effects are mediated by ERK1/2 and AKT-dependent survival pathways, and galectin-1 inhibition induces of G2/M cell cycle arrest (58). Immunomodulatory activities are well documented for galectin-1, which acts as a suppressor of T cell anti-tumor immunity (59), enhances regulatory monocyte/macrophage subsets (60), promotes tolerogenic dendritic cells and in certain scenarios has been shown to trigger damage-associated molecular pattern (DAMP) pathway activation (61). Galectin-1 is a transcriptional target of NFκB, and its expression and release are enhanced via TNF signaling via NFκB (62). We show that a feedback loop exists wherein expanded basophil, mast cell, megakaryocyte and stromal cell subsets induce a self-reinforcing pro-inflammatory niche and galectin-1 expression, fueling inflammation and fibrosis (Fig. 7J). Targeting galectin-1 using small molecule glycan inhibitors, natural polysaccharides, peptides (OTX008) or anti-galectin-1 monoclonal antibodies may counteract fibrosis and also the immunomodulation that occurs in myeloid neoplasms (61, 63).

Ongoing work will be aimed at determining the mechanisms of action for galectin-1 in myeloid neoplasms, further validating the efficacy of galectin-1 targeting in additional disease models, and identifying the most clinically-tractable targeting modality. In addition, while significantly increased expression of *LGALS1* was seen in AP/BP-MPN and a clear correlation found between high expression of *LGALS1* and poor survival in *de novo* AML associated with inflammatory signalling, further work is required to establish a mechanistic role for galectin-1 in leukemic progression. Collectively, the data presented here confirm a role for galectin-1 as a mediator of pathobiology in myeloid malignancies and worthy of further exploration as a therapeutic target that has the potential to modify the disease course. The road-map of cellular interactions in myelofibrotic bone marrow has broad implications for other hematological malignancies, cancer-associated inflammation and non-malignant fibrotic disorders.

## Materials and Methods

### Animal Studies

All mice were bred and maintained in accordance with UK Home Office regulations, and experiments were conducted in accordance with approvals from the University of Oxford Animal Welfare and Ethical Review Body (project license P22FF90EE8). For transplantation experiments, C57BL/6J (CD45.2) mice were used as donors, and CD45.1 mice were used as recipient mice and as a source of competitor cells.

### MPL^W515L^ murine model

The MPL^W515L^ murine model was established as previously described (14). Details about the procedure are reported in the Supplemental Material.

### *In vivo* treatments

The anti-galectin-1 neutralising antibody (mAb3) was produced as previously reported (36). Seven days after transplantation, either an isotype IgG control antibody or mAb3 were administered twice weekly for 14 days via intraperitoneal (i.p.) injections, at alternating locations at 25 mg/kg. For comparison with a standard-of-care agent, we treated a subset of mice with fedratinib (MedChemExpress). In this cohort, seven days after transplantation, PBS or fedratinib (120mg/kg) (64) were administered 5 days per week for 2 weeks via once daily oral gavage treatment.

### Banking and processing of human samples

Patients and healthy donors provided written informed consent in accordance with the Declaration of Helsinki for sample collection, tissue banking and use in research under the Informed study (Investigating the genetic and cellular basis of sporadic and Familial Myeloid Disorders; IRAS ID: 199833; REC reference: 16/LO/1376; PI: Prof AJ Mead). Cryopreserved bone marrow mononuclear cells isolated by density gradient centrifugation using Ficoll-Paque Premium (Sigma Aldrich) were cryopreserved in FCS with 10% DMSO (Sigma Aldrich) and thawed and processed by warming briefly at 37°C, gradually diluted into RPMI-1640 (Gibco), supplemented with 10% FCS and 0.1mg/mL DNase I (Sigma), centrifuged at 500G for 5 minutes and washed in FACS buffer (PBS + 2mM EDTA + 5% FCS).

Primary human BMSCs were isolated as previously reported (65). Briefly, cryopreserved mononuclear cells from bone marrow aspirates were thawed and cultured in αMEM (Gibco) supplemented with 10% FBS for 3-4 days. Subsequently non-adherent cells were removed, whereas stromal cells were selected by their adherence to plasticware.

For analysis of galectin-1 mRNA expression in platelets, published platelet RNA sequencing data was analyzed (38). Eligibility criteria included age ≥18 years and Stanford MPN clinic diagnosis of essential thrombocythemia, polycythemia vera or myelofibrosis (defined using the consensus criteria at the time of this study). For healthy controls, blood was collected from twenty-one asymptomatic adult donors selected at random from the Stanford Blood Center. All donors provided written consent for genetic research. For both MPN patients and healthy controls, blood was collected into acid citrate-dextrose (ACD, 3.2%) sterile yellow-top tubes (Becton, Dickinson and Co.) and platelets were isolated by established (66–69) purification protocols. Blood was processed within 4 h of collection for all samples. The time from whole blood collection to platelet isolation was similar between healthy donors and MPN patients.

### Murine stromal cell isolation

Murine bone marrow stromal cells (BMSCs) were isolated as previously described (15). In brief, long bones were flushed and the central bone marrow was digested with 2mg/ml Collagenase IV (Thermo Fisher Scientific) at 37°C for 20min. The bones were cut or crushed and digested with 3mg/ml Collagenase I (Thermo Fisher Scientific) at 37°C for 1.5h. Cells were pooled, treated for 10 min with NH4Cl solution (STEMCELL Technologies), washed with PBS and CD45 negative cells were enriched using mouse CD45 microbeads (Miltenyi Biotec) depletion of CD45+ cells.

### Fluorescent activated cell sorting (FACS) for single cell RNA sequencing

Details about the isolation of stromal cells and the different hematopoietic cells populations are reported in Supplemental material.

Samples were then processed according to the 10x protocol using the Chromium Single Cell 3’ library and Gel Bead Kits version 3 and 3.1 (10x Genomics). Pre-amplified cDNA was used for library preparation, multiplexed, and sequenced on a Novaseq 6000, aiming to obtain > 50,000 reads per cell.

### Fluorescent activated cell sorting (FACS) for low-input proteomics

BM stromal cells from n = 4 GFP control mice were isolated as reported below and then stained at RT for 30 minutes with the following antibody panel: AF700 anti-CD45, PerCP Cy5.5 anti-TER-119, BV605 anti-Sca-1, PE Cy7 anti-CD31, Biotin anti-LEPR/Streptavidin PE CF594, APC anti-PDGFRA, and PE anti-CD71. DAPI was used as a live-dead marker. Fibroblasts were defined as Ter-119^-^CD71^-/low^-CD45^-^PDGFRA^+^, Endothelial cells (ECs) as Ter-119^-^CD71^-/low^-CD45^-^PDGFRA^-^Sca-1^+^CD31^+^ and leptin receptor positive mesenchymal stromal cells (LEPR+ MSC) as Ter-119^-^CD71^-/low^-CD45^-^PDGFRA^-^CD31^-^LEPR^+^. For each population, 500 cells per well were sorted into 384-well plates (Eppendorf twin.tec 384 LoBind) containing 1µl of lysis buffer (0.2% DDM, 80mM TEAB). After sorting, the plates were briefly spun, snap-frozen on dry ice, and boiled for 5 minutes at 95°C. Subsequently, the plates were cooled on ice, briefly spun again, and stored at -80°C until further analysis.

### Fibroblast to myofibroblast transition assay using human BMSC

BMSCs were seeded into collagen-treated 348 wells imaging plates (Corning cat # 356667) at 5000 cells/well and cultured for 24h in αMEM (Gibco) supplemented with 0.3% FBS, 200µM Hepes (Gibco), 50μM ß-mercaptoethanol (Gibco) and 30µg/mL ascorbic acid (Sigma Aldrich). Then media was replaced, and cells were cultured for 72h in FMT media in presence or absence of 10ng/ml TGFβ (Biolegend), 4μM OTX008 (MedChemExpress), or 20μM SB431542 (MedChemExpress). At the end of the assay, cells were fixed in cold methanol, blocked with 6% FBS in PBS and then stained for 1h with the primary antibodies for aSMA (Sigma Aldrich, 1:500) and Collagen 1 IgG1 (Sigma Aldrich, 1:4000) for 1h at RT. After that, wells were washed for 3 times with PBS and incubated with the secondary antibody Alexa488 (Thermo Scientific, 1:2000) or Alexa 568 (Thermo Scientific, 1:1000) – at RT for 2h. DRAQ5 was used to stain the nuclei. Images were acquired using the IN Cell Analyzer 6000 (GE Healthcare).

For image analysis, we used a bespoke imaging analysis program to automate calculation of the mean intensity of fluorescence for collagen 1 per well. The program takes paired grey-scale images for nuclei and collagen 1, counts the number of cells (nuclei) using edge detection and calculates the mean intensity for collagen 1 staining per cell. The source code is available at https://github.com/QCheng91/ImmunofluorescentDetection.

### Intracellular flow of basophils for IL4 and TNF

For analysis of basophils differentiated *in vitro*, cKit+ murine stem/progenitor cells were transduced with *MPL^W515L^-GFP* or control-GFP vectors and cultured as previously described (70) at a density of 2 x 10^6^ cells/ml in IMDM containing 10% FBS, 1% Pen/Strep and 10% conditioned media from BHK/MKL cells (as a source of SCF), 150 uM monothioglycerol (Sigma) and 10 ng/ml m-IL3 (PeproTech). Every 2 to 3 days cells were transferred into new media, and basophils analysed on day 7 of the differentiation (defined as live cells, GFP+, FcER1a^+^/CD117^-^). For *ex vivo* analysis of bone marrow basophils, flushed bone marrow cells were gated as 7AADneg, GFP^+^, lineage (B220, CD3, CD11b, Gr-1, Ter-119) neg, FcER1a^+^ CD117neg. Expression of IL4 and TNF was analysed by intracellular flow cytometry. In brief, cells were transferred into 96-well plates and incubated with 1 µl/ml BD GolgiPlug for 4 hours at 37° Celsius. Following live dead staining with Zombi Red (BioLegend) and subsequent staining of cell surface antigens (CD117, SiglecF and FcER1a), cells were then fixed and permeabilised using the BD Cytofix/Cytoperm™ Fixation/Permeabilization Kit, and IL4 and TNF antibodies were added (Table S1). Samples were acquired using the LSR Fortessa X-20 (BD) and data were analysed with FlowJo.

### Fibrosis assay with human bone marrow organoids

Human bone marrow organoids were derived from human induced pluripotent stem cells (hiPSCs) as previously described (42). At day 18 of the differentiation protocol, organoids were cultured for 72h with 10ng/ml TGFβ (Biolegend) in presence of 30μg/ml ascorbic acid (Sigma Aldrich), followed by 72h in the presence or absence of 10 ng/ml TGFβ, 4μM or 8μM OTX008 (MedChemExpress), or 20μM SB431542 (MedChemExpress). After treatment, organoids were collected for either fixation and imaging, or digested with 5mg/ml Collagenase II in HBSS (Sigma Aldrich) for 20min at 37°C with gentle agitation to perform RNA extraction and qRT-PCR.

For imaging, organoids were fixed in 4% PFA for 30 minutes with gentle agitation before a series of PBS washes. Washed samples were blocked in 2% goat serum, 1% BSA, Triton X100 (Sigma Aldrich), 250µL Tween-20 (Sigma Aldrich), and 500 µL sodium deoxycholate (w/v) (Sigma Aldrich) in PBS before labelling in blocking buffer with the same antibodies used in the FMT assay. Labelled samples were then embedded in low molecular weight agarose and subject to a serial dehydration and ethyl cinnamate clearance before imaging on a Zeiss LSM 880 AiryScan confocal (42).

For image analysis, images were processed using ImageJ/Fiji. Z stacks were subject to a maximum intensity projection before denoising and background subtraction (rolling ball). Regions of interest were drawn around organoids, and the fluorescence intensity calculated per organoid.

### RNA extraction and qRT-PCR

Total RNA was isolated using the Qiagen Mini RNA isolation kit (Qiagen) and cDNA was prepared using EvoScript Universal cDNA Master (Roche) according to manufacturers’ instructions. Quantitative real time PCR (qRT-PCR) was performed on a StepOne plus machine (Applied Biosystem) using the 2-ΔCt analysis method. Details of the TaqMan gene expression assays (Thermofisher Scientific) used are available in Supplemental Table 1.

### Immunohistochemistry

Mouse bones and spleens were fixed using 4% Formaldehyde solution and processed for IHC, or hematoxylin/eosin stain, or reticulin staining. Human bone marrow biopsy samples were fixed in 10% neutral buffered formalin prior to decalcification in 10% EDTA for 48 hrs. Histopathological diagnosis was carried out according to the WHO classification (71). Galectin1 staining was performed using 1:400 Anti-Galectin-1 antibody (Abcam). Antigens were visualized using diaminobenzidine (DAB) as chromogen.

### High throughput single cell transcriptomics sequencing (10x Genomics)

FACS sorted cells from each sample were processed according to the 10x Genomics protocol using the Chromium Single Cell 3′ library and Gel Bead Kits v3 (10x Genomics).

### Low-input proteomics

Low input proteomic on the different cells populations was performed as previously reported (72). Briefly, protein digestion was conducted overnight at 37 °C by adding Trypsin (Promega) at a concentration of 10 ng/uL in 100 mM TEAB (pH 8.5) and subsequently stopped by the addition of 4 % (v/v) trifluoroacetic acid (TFA). Prior to mass spectrometry analysis, digested samples were loaded on Evotip pure (Evosep) columns for online desalting following the manufacturers recommendations. Chromatographic separation of peptides derived was conducted over a 58-minute gradient on an EvosepOne UHPLC system (Evosep) connected to a 15 cm Aurora Elite TS (Ion Opticks) maintained at 50 °C. Following ionization, MS-spectra were collected using a Orbitrap Eclipse Tribrid mass spectrometer equipped with FAIMS Pro interface (Thermo Scientific) and operated in positive mode with a compensation voltage of -45 V. MS1 spectra were collected in the Orbitrap at a resolution 120k and a mass range of 400 to 1000 Th. Automatic gain control (AGC) was set at 300 % and a maximum injection time set to 246 ms. Fragmentation of precursor ions was achieved through higher energy collisional dissociation (HCD) using a normalized collision energy of 33%. Data-independent acquisition was conducted in the Orbitrap at the same resolution utilizing loop control set to 12 spectra per loop and isolation windows of 17 Th over a mass range of 200 to 1200 Th resulting in 36 windows across all looped cycles. For this, AGC was set at 1000 % and the maximum injection time was configured to automatic.

### Mass spectrometry data analysis

Obtained .raw files were processed with Spectronaut (v.18) in directDIA mode using standard settings with the following modifications: Quantity MS level was changed to MS1 and Carbamidomethylation of cysteines was removed as fixed modification. Protein quantification matrices were then exported and further downstream analysis. Log normalization was performed to stabilize the variance and reduce skewness throughout the dataset. The data was subsequently scaled to a fixed range (0 – 1) unsing min-max normalization to ensure that all protein expression levels were on a comparable scale. This dual normalization approach facilitated the precise comparison of protein expression levels across the different samples. A Wilcoxon test was conducted to ascertain the differences in protein abundance between cell types after normalization steps.

### Single cell transcriptomics analysis

We used CellRanger software version 3.0.1 (10x Genomics) to obtain cell counts using “cellranger count” command to align the reads to the mm10 genome to identify cell barcodes and generate the expression matrix. Single-cell RNA sequencing analysis was performed using SingCellaR software (v1.2.0)42. Briefly, we analyzed the cells that passed the following QC parameters: min UMI counts > 1,000 and ≤ maximum UMIs; min number of detected genes > 500 and ≤ maximum number of detected genes and genes expressed at least in 10 cells and 10% as the mitochondria cut-off. Then, individual objects were integrated and highly variable genes were identified using the ‘get_variable_genes_by_fitting_GLM_model’ function, retaining 1536 highly variable genes for stromal populations and 1306 genes for haematopoietic populations respectively for downstream analysis. Stromal and haematopoietic populations were analysed separately to ensure that all the cells were correctly clustered and annotated. Principal component analysis (PCA) was performed using the top 50 PCs and the Harmony method was used (‘runHarmony’ function in SingCellaR) on the top 30 PCs to integrate the datasets and correct the batch effects for downstream analyses including UMAP analysis, Louvain clustering and cell type annotations. Cell types were annotated by combining three strategies: 1) annotation of the clusters by canonical marker genes; 2) implementation the semi-automatic annotation method in SingCellaR; 3) visualization of multiple lineages genesets on top of the UMAP plot using ‘plot_umap_label_by_multiple_gene_sets’ function.

Hematopoietic ‘contamination’ removal in the stromal samples

We aimed to investigate the gene expression profiles of stromal cells in the bone marrow, however, a key challenge was the potential ‘contamination’ of hematopoietic cells in the stromal samples. To solve this problem, we obtained a list of cell-type-specific marker genes for hematopoietic cells and stromal cells from previous studies (15–17)and then performed two rounds of removal of hematopoietic cell clusters.

### Doublet removal

We implemented a two-step doublet removal method to ensure data quality. First, we applied the Scrublet algorithm to each individual sample as per the documentation. Secondly, the doublets were projected onto the UMAP plot of integrated objects to visualize the doublets. We then examined the expression of multiple genes lineages using the ‘plot_umap_label_by_multiple_gene_sets’ function in SingCellaR to confirm their cellular identities. We identified two additional doublet clusters in hematopoietic populations (Fig. S1C). We removed the doublets identified by Scrublet (279 cells in hematopoietic populations and 89 cells in stromal populations) and the doublet clusters (374 cells in hematopoietic populations, 0 cell in stroma population). Following doublet removal, the objects were used for differentially expressed genes analysis by standard SingCellaR workflow.

### Symphony analysis

We applied Symphony (22) to overlay published healthy and myelofibrotic mouse bone marrow stroma and hematopoietic scRNAseq datasets onto our datasets. We first used ‘buildReference’ function to build the reference UMAP plots using the control mice only stroma, control + MPL^W515L^ stroma and all hematopoietic cells in our study and colored by the annotated cell types. Then we used ‘mapQuery’ function to perform dataset projection.

### Differential abundance test

We performed differential abundance testing using the MiloR software (v0.1.0) (73) between Control and MPL^W515L^ mice for MNCs in the hematopoietic populations and stroma cells respectively. MiloR is an R package designed for differential abundance testing in single-cell transcriptomics analysis. We created a miloR object using the ‘Milo’ function. 30 dimensions were used to calculated neighborhood distance. We then used the ‘buildNhoodGraph’ function to perform differential abundance testing between different clusters. The differential abundance test generated a list of significantly differentially abundant fractions with their respective P-values and fold changes.

### Extra cellular matrix (ECM), chemokine and Niche Supporting Factor (NSF) scores

We analyzed the expression of ECM factors from a previously published database (25)in each single cell by defining an ECM score using the total expression of expressed ECM genes divided by total expression of all the genes within a cell. The expressed ECM genes were defined as having a minimum count of 50 UMIs across all cells in hematopoietic populations or stromal populations. For the chemokine and NSF gene scores, we curated the genes from published studies (Supplemental Table 4) and analyzed the NSF gene score as described for the ECM score.

### Gene set enrichment analysis (GSEA)

We used the ‘Run_fGSEA_analysis’ function to compare two groups of cells in SingCellaR. Genes were pre-ranked using the function ‘identifyGSEAPrerankedGenes’.

### Differentiation state analysis

We applied CytoTrace (74) on the EBM cluster to investigate the differentiation state. Briefly, we extracted the expression matrix (counts) from the SingcellaR object and then used the function ‘CytoTRACE’ to calculate the CytoTRACE score for each cell. This score implies the differentiation state for each cell. To visualize the result on a 2D plot, we plotted a 2D plot with UMAP embeddings of the EBM object.

### scTour anlaysis

scTour (75) is a method for dissecting cellular dynamics. We extracted the expression matrix (counts), metadata and UMAP from the SingCellaR fibroblast object and transformed to AnnData. Then we counted the number of genes detected in each cell and trained the model using negative binomial distribution as the loss function to get the pseudotime variable for each cell. We further counted the latent representations and transcriptomic vector field and projected it onto a UMAP embedding.

### Cell-cell interaction analysis

We applied CellChat (32) to analyze the cellular interactions. We first built a customized ligand-receptor database followed by the tutorial and merged the bone marrow stroma and hematopoietic cells into one object and normalized the counts. The objects were then split to ‘Control’ and ‘MPL^W515L^’ groups and the cellular interactions were analyzed separately. To compare the differential interactions between ‘Control’ and ‘MPL^W515L^’ groups, we used the ‘mergeCellChat’ function to merge the ‘Control’ and ‘MPL^W515L^’ Cellchat objects and compared the interaction by the ‘compareInteractions’ function. We further applied the ‘netVisual_heatmap’ function to show the differentially expressed number of interactions of MSC, iFibs, other Fibs, EBM and MK. Selected differentially expressed ligand-receptor pairs were shown in Fig. 5E using the ‘netVisual_bubble’ function.

### Continuous Indexing of Fibrosis (CIF) scores assessment

A Learning to Rank (LTR) strategy known as RankNet (76)is used to assess the severity of fibrosis within and between myelofibrosis grades (MFs). The RankNet model predicts the order in which features are ranked according to their severity. A Convolutional Neural Network (CNN) is used as a feature extractor for a model to learn to rank because of its high performance in many applications, especially in medical image analysis (77). Therefore, the Ranking-CNN model was developed by combining RankNet with a CNN (37). The trained model then outputs the score. This was used as a reference for fibrosis severity which is called Continuous Indexing of Fibrosis (CIF) scores (37). CIF scores approach 1 when the sample is more fibrotic. To visualize the spectrum of the fibrosis within the sample, a map of fibrosis severity is generated using CIF scores.

### Galectin-1 quantification

To quantify galectin-1 expression, we identified pixels with positive staining in the tissue. We applied stain deconvolution (78) to computationally separate the galectin-1 stain channel from the DAPI stain channel and employed stain normalization (79) to address staining variability across different sample batches. To identify galectin-1-positive pixels, we obtained the galectin1 pixel intensity distribution from all tissue samples and used the Otsu method (80) to determine an appropriate cut-off. A heatmap showing the level and variation of galectin1 expression within the same tissue is generated by calculating the ratio of galectin1-positive pixels in multiple small tissue areas (256-by-256 microns).

### Analysis of *LGALS1* in human *de novo* AML and leukemic transformation of MPN

TCGA AML patient survival data and gene expression values were retrieved as previously described (81). In total, 132 patients with survival and gene expression data were available and included for survival and gene set enrichment analysis (GSEA) (82). To interrogate *LGALS1* expression in patients with blast phase MPN, we explored published RNA-sequencing data of CD34+ HSPCs and total mononuclear cells from patients with accelerated/blast phase MPN (AP/BP-MPN, n = 10) and healthy donors (n = 5) (46).

More details about survival analysis and GSEA are available in Supplemental materials.

### Statistical analysis

Statistical analysis was performed with Prism9.0. Details of statistical tests are included in figure legends.

## Supplementary Material

adj7552_SupplementalMaterial_FINAL ACCEPTED VERSION.pdf

## Data Availability

All raw and processed sequencing data generated in this study have been submitted to the NCBI Gene Expression Omnibus (GEO; https://www.ncbi.nlm.nih.gov/geo/) under accession number GSE228995(Reviewer token: sjkdskyilfefjen). The corresponding sample information is contained in Suppl. Table 5. All code will be deposited at Github: https://github.com/guanlinW/Mouse-Bone-Marrow-10X. Materials and reagents used in this study are listed in Table S1.

## One Sentence Summary

Unravelling the cellular landscape of myelofibrosis reveals novel drivers of inflammation and galectin-1 as a clinically actionable target.
