# Supplementary material for: A pro-inflammatory stem cell niche drives myelofibrosis through a targetable galectin-1 axis
Source: Sci Transl Med. Author manuscript; Available in PMC 2024 Nov 4. (PMC7616771; doi:10.1126/scitranslmed.adj7552)
Supplement: adj7552_SupplementalMaterial_FINAL ACCEPTED VERSION.pdf [file EMS199448-supplement-adj7552_SupplementalMaterial_FINAL_ACCEPTED_VERSION_pdf.pdf]

Materials and Methods

Mpl^{W515L} murine model

To produce retroviral supernatant, transient co-transfection of HEK293T cells with pCL_Ampho retroviral packaging vector and the MSCV-*MplW515*-IRES-EGFP were performed using PEI Pro (Polysciences, Inc) according to manufacturer's guidelines. The MSCV-IRES-EGFP empty vector was used as control. The retroviral supernatant was collected 48h or 72h post transfection, filtered and stored at -80°C.

For each experiment, bone marrow cells were harvested from 7-8 weeks old C57BL/6OlaHsd (CD45.2) female mice by isolating and crushing the bones. Bone marrow cKit+ cells were enriched using mouse CD117 microbeads (Miltenyi Biotec), and pre-stimulated in IMDM (Gibco) with 10% FBS (Sigma Aldrich), 100 ng/ml stem cell factor (SCF) (Peprotech), 20 ng/ml Flt-3 ligand (Flt3L) (Peprotech), 20 ng/ml interleukin (IL)-3 (Peprotech) and 20 ng/ml IL-11 (Peprotech) for 24 hours. 12-well tissue culture plates were coated with retronectin (Takarabio) and left overnight at 4°C. After 24 hours, retronectin-coated wells were pre-loaded with GFP only or *MPL*^{W515L}-GFP virus supernatant by centrifugation at 2000rpm for 60 min at 4 °C. Meanwhile, the pre-stimulated cKit+ cells were harvested and resuspended in IMDM with 10% FBS, 200 ng/ml SCF, 40 ng/ml Flt-3 Flt3L, 40 ng/ml IL-3 and 40 ng/ml IL-11. The virus supernatant was removed from the wells, and the pre-stimulated cKit+ cells were added, together with the same volume of GFP or *MPL*^{W515L} virus supernatant into the preloaded plate. After 24 hours of transduction, cells were washed 3 times and intravenously injected along with wild-type CD45.1 B6.SJL-Ptprca Pepcb/BoyJ bone marrow cells into lethally irradiated (2 × 4.5 Gy [450 rad]), CD45.1 B6.SJL-Ptprca Pepcb/BoyJ recipient mice. Animals were humanely killed when they had palpable splenomegaly or were moribund. Peripheral blood was collected from the tail vein or via cardiac puncture into EDTA-coated microvettes (Sarstedt) and diluted five times in PBS prior to analysis on a Pentra ES 60 Cell Counter (HORIBA ABX SAS). Mice were

monitored and culled if the body weight loss was more than 15%. For the MPN-free survival curve analysis, MPN was defined as white blood cell count ≥ $60*10^9$/L, HGB ≤ 10 g/dL or ≥ 15 g/dL, platelet count ≥ $1600*10^9$/L or spleen size > 1g.

Immunohistochemistry

Mouse bones and spleens were fixed using 4% Formaldehyde solution and processed for IHC, or hematoxylin/eosin stain, or reticulin staining. Human bone marrow biopsy samples were fixed in 10% neutral buffered formalin prior to decalcification in 10% EDTA for 48 hrs. Histopathological diagnosis was carried out according to the WHO classification (*65*). Galectin1 staining was performed using 1:400 Anti-Galectin-1 antibody (Abcam). Antigens were visualized using diaminobenzidine (DAB) as chromogen.

Murine stromal cells isolation

Murine bone marrow stromal cells (BMSCs) were isolated as previously described (*15*). In brief, long bones were flushed and the central bone marrow was digested with 2mg/ml Collagenase IV (Thermo Fisher Scientific) at 37°C for 20min. The bones were cut or crushed and digested with 3mg/ml Collagenase I (Thermo Fisher Scientific) at 37°C for 1.5h. Cells were pooled, treated for 10 min with NH4Cl solution (STEMCELL Technologies), washed with PBS and CD45 negative cells were enriched using mouse CD45 microbeads (Miltenyi Biotec) depletion of CD45+ cells.

Fluorescent activated cell sorting (FACS) for single cell RNA sequencing

To capture stromal cells, CD45+ cell bead-depleted cells were incubated with Fc block for 15 minutes at 4°C. Cells were then stained with anti-mouse CD45, anti-mouse hematopoietic lineage marker cocktail (CD11b, CD3e, CD19, B220, Gr1), anti-mouse Ter119 and anti-mouse CD71 for 20 min at 4°C in PBS 1% FCS 2mM EDTA (Thermo Fisher Scientific), antibody details in Supplemental Table 1. DAPI (Sigma Aldrich) was added prior to analysis and sorting as a cell viability dye. Stromal cells were identified as

DAPI-CD45-Lin- Ter119- CD71- and were sorted into 2µL PBS/0.05% BSA (non-acetylated) on an Aria™ Fusion Cell Sorter (BD Biosciences).

To capture hematopoietic cells, bone marrow cells obtained by crushing the femurs, tibias and cristae were suspended in PBS + 1% BSA and incubated with Fc block for 15 minutes at 4°C. Antibody staining was performed using the antibodies listed in Supplemental Table 1 for 20 min at 4°C in PBS + 1% FCS + 2mM EDTA and sorted into 2µL PBS + 0.05% BSA (non-acetylated) on an Aria™ Fusion Cell Sorter (BD Biosciences). The following populations were sorted: (i) Total viable mononuclear cells (DAPI- GFP+); (ii) HSC/MPP or lineage negative, cKit+ cells (DAPI- GFP+ Lin- CD117+ cells) and (iii) CD41+ cells (DAPI- GFP+ CD41+). The CD41+ fraction was sorted using a 130nm nozzle. All other hematopoietic and stromal cell populations were sorted using a 100nm nozzle. Cell number and volume was adjusted prior to loading onto the 10x Chromium Controller for droplet generation.

High throughput single cell transcriptomics sequencing (10x Genomics)

FACS sorted cells from each sample were processed according to the 10x Genomics protocol using the Chromium Single Cell 3' library and Gel Bead Kits v3 (10x Genomics). Briefly, cells and reagents were prepared and loaded onto the chip and into the Chromium Controller for droplet generation. RT was conducted in the droplets and cDNA recovered through demulsification and bead purification. Pre-amplified cDNA was used for library preparation, multiplexed and sequenced aiming to obtain > 50,000 reads per cell.

Single cell transcriptomics analysis

We used CellRanger software version 3.0.1 (10x Genomics) to obtain cell counts using "cellranger count" command to align the reads to the mm10 genome to identify cell barcodes and generate the expression matrix. Single-cell RNA sequencing analysis was performed using SingCellaR software (v1.2.0)(*66*) . Briefly, we analyzed the cells that passed the following QC parameters: min UMI counts >

1,000 and £ maximum UMIs; min number of detected genes > 500 and £ maximum number of detected genes and genes expressed at least in 10 cells and 10% as the mitochondria cut-off. Then, individual objects were integrated and highly variable genes were identified using the 'get_variable_genes_by_fitting_GLM_model' function, retaining 1536 highly variable genes for stromal populations and 1306 genes for haematopoietic populations respectively for downstream analysis. Stromal and haematopoietic populations were analysed separately to ensure that all the cells were correctly clustered and annotated. Principal component analysis (PCA) was performed using the top 50 PCs and the Harmony method was used ('runHarmony' function in SingCellaR) on the top 30 PCs to integrate the datasets and correct the batch effects for downstream analyses including UMAP analysis, Louvain clustering and cell type annotations. Cell types were annotated by combining three strategies: 1) annotation of the clusters by canonical marker genes; 2) implementation the semi-automatic annotation method in SingCellaR; 3) visualization of multiple lineages genesets on top of the UMAP plot using 'plot_umap_label_by_multiple_gene_sets' function.

Hematopoietic 'contamination' removal in the stromal samples

We aimed to investigate the gene expression profiles of stromal cells in the bone marrow, however, a key challenge was the potential 'contamination' of hematopoietic cells in the stromal samples. To solve this problem, we obtained a list of cell-type-specific marker genes for hematopoietic cells and stromal cells from previous studies (*15-17*) and then performed two rounds of removal of hematopoietic cell clusters.

Doublet removal

We implemented a two-step doublet removal method to ensure data quality. First, we applied the Scrublet algorithm to each individual sample as per the documentation. Secondly, the doublets were projected onto the UMAP plot of integrated objects to visualize the doublets. We then examined the

expression of multiple genes lineages using the ‘plot_umap_label_by_multiple_gene_sets’ function in SingCellaR to confirm their cellular identities. We identified two additional doublet clusters in hematopoietic populations (Fig. S1C). We removed the doublets identified by Scrublet (279 cells in hematopoietic populations and 89 cells in stromal populations) and the doublet clusters (374 cells in hematopoietic populations, 0 cell in stroma population). Following doublet removal, the objects were used for differentially expressed genes analysis by standard SingCellaR workflow.

Symphony analysis

We applied Symphony (*22*) to overlay published healthy and myelofibrotic mouse bone marrow stroma and hematopoietic scRNAseq datasets onto our datasets. We first used ‘buildReference’ function to build the reference UMAP plots using the control mice only stroma, control + *Mpl*^{W515L} stroma and all hematopoietic cells in our study and colored by the annotated cell types. Then we used ‘mapQuery’ function to perform dataset projection.

Differential abundance test

We performed differential abundance testing using the MiloR software (v0.1.0) (*67*) between Control and *Mpl*^{W515L} mice for MNCs in the hematopoietic populations and stroma cells respectively. MiloR is an R package designed for differential abundance testing in single-cell transcriptomics analysis. We created a miloR object using the ‘Milo’ function. 30 dimensions were used to calculated neighborhood distance. We then used the ‘buildNhoodGraph’ function to perform differential abundance testing between different clusters. The differential abundance test generated a list of significantly differentially abundant fractions with their respective P-values and fold changes.

Extra cellular matrix (ECM), chemokine and Niche Supporting Factor (NSF) scores

We analyzed the expression of ECM factors from a previously published database (*25*)in each single cell by defining an ECM score using the total expression of expressed ECM genes divided by total

expression of all the genes within a cell. The expressed ECM genes were defined as having a minimum count of 50 UMIs across all cells in hematopoietic populations or stromal populations. For the chemokine and NSF gene scores, we curated the genes from published studies (Supplemental Table 4) and analyzed the NSF gene score as described for the ECM score.

Gene set enrichment analysis (GSEA)

We used the 'Run_fGSEA_analysis' function to compare two groups of cells in SingCellaR. Genes were pre-ranked using the function 'identifyGSEAPrerankedGenes'.

Differentiation state analysis

We applied CytoTrace (*68*) on the EBM cluster to investigate the differentiation state. Briefly, we extracted the expression matrix (counts) from the SingcellaR object and then used the function 'CytoTRACE' to calculate the CytoTRACE score for each cell. This score implies the differentiation state for each cell. To visualize the result on a 2D plot, we plotted a 2D plot with UMAP embeddings of the EBM object.

scTour anlaysis

scTour (*69*) is a method for dissecting cellular dynamics. We extracted the expression matrix (counts), metadata and UMAP from the SingCellaR fibroblast object and transformed to AnnData. Then we counted the number of genes detected in each cell and trained the model using negative binomial distribution as the loss function to get the pseudotime variable for each cell. We further counted the latent representations and transcriptomic vector field and projected it onto a UMAP embedding.

Cell-cell interaction analysis

We applied CellChat (*32*) to analyze the cellular interactions. We first built a customized ligand-receptor database followed by the tutorial and merged the bone marrow stroma and hematopoietic cells

into one object and normalized the counts. The objects were then split to 'Control' and 'MPL^{W515L}' groups and the cellular interactions were analyzed separately. To compare the differential interactions between 'Control' and 'MPL^{W515L}' groups, we used the 'mergeCellChat' function to merge the 'Control' and 'MPL^{W515L}' Cellchat objects and compared the interaction by the 'compareInteractions' function. We further applied the 'netVisual_heatmap' function to show the differentially expressed number of interactions of MSC, iFibs, other Fibs, EBM and MK. Selected differentially expressed ligand-receptor pairs were shown in Fig. 5E using the 'netVisual_bubble' function.

Fluorescent activated cell sorting (FACS) for low-input proteomics

BM stromal cells from n = 4 GFP control mice were isolated as reported below and then stained at RT for 30 minutes with the following antibody panel: AF700 anti-CD45, PerCP Cy5.5 anti-TER-119, BV605 anti-Sca-1, PE Cy7 anti-CD31, Biotin anti-LEPR/Streptavidin PE CF594, APC anti-PDGFRA, and PE anti-CD71. DAPI was used as a live-dead marker. Fibroblasts were defined as Ter-119^{-}CD71$^{-/low}$-CD45^{-}PDGFRA^{+}, Endothelial cells (ECs) as Ter-119^{-}CD71$^{-/low}$-CD45^{-}PDGFRA^{-}Sca-1^{+}CD31^{+} and leptin receptor positive mesenchymal stromal cells (LEPR+ MSC) as Ter-119^{-}CD71$^{-/low}$-CD45^{-}PDGFRA^{-}CD31^{-}LEPR^{+}. For each population, 500 cells per well were sorted into 384-well plates (Eppendorf twin.tec 384 LoBind) containing 1µl of lysis buffer (0.2% DDM, 80mM TEAB). After sorting, the plates were briefly spun, snap-frozen on dry ice, and boiled for 5 minutes at 95°C. Subsequently, the plates were cooled on ice, briefly spun again, and stored at -80°C until further analysis.

Low-input proteomics

Low input proteomic on the different cells populations was performed as previously reported (*72*). Briefly, protein digestion was conducted overnight at 37 °C by adding Trypsin (Promega) at a concentration of 10 ng/uL in 100 mM TEAB (pH 8.5) and subsequently stopped by the addition of 4 % (v/v) trifluoroacetic acid (TFA). Prior to mass spectrometry analysis, digested samples were loaded on Evotip pure (Evosep) columns

for online desalting following the manufacturers recommendations. Chromatographic separation of peptides derived was conducted over a 58-minute gradient on an EvosepOne UHPLC system (Evosep) connected to a 15 cm Aurora Elite TS (Ion Opticks) maintained at 50 °C. Following ionization, MS-spectra were collected using a Orbitrap Eclipse Tribrid mass spectrometer equipped with FAIMS Pro interface (Thermo Scientific) and operated in positive mode with a compensation voltage of -45 V. MS1 spectra were collected in the Orbitrap at a resolution 120k and a mass range of 400 to 1000 Th. Automatic gain control (AGC) was set at 300 % and a maximum injection time set to 246 ms. Fragmentation of precursor ions was achieved through higher energy collisional dissociation (HCD) using a normalized collision energy of 33%. Data-independent acquisition was conducted in the Orbitrap at the same resolution utilizing loop control set to 12 spectra per loop and isolation windows of 17 Th over a mass range of 200 to 1200 Th resulting in 36 windows across all looped cycles. For this, AGC was set at 1000 % and the maximum injection time was configured to automatic.

Mass spectrometry data analysis

Obtained .raw files were processed with Spectronaut (v.18) in directDIA mode using standard settings with the following modifications: Quantity MS level was changed to MS1 and Carbamidomethylation of cysteines was removed as fixed modification. Protein quantification matrices were then exported and further downstream analysis. Log normalization was performed to stabilize the variance and reduce skewness throughout the dataset. The data was subsequently scaled to a fixed range (0 – 1) unsing min-max normalization to ensure that all protein expression amounts levels were on a comparable scale. This dual normalization approach facilitated the precise comparison of protein expression across the different samples. A Wilcoxon test was conducted to ascertain the differences in protein abundance between cell types after normalization steps.

Intracellular flow of basophils for IL4 and TNF

For analysis of basophils differentiated *in vitro*, cKit+ murine stem/progenitor cells were transduced with *MPL*^{W515L}*-GFP* or control-GFP vectors and cultured as previously described (*70*) at a density of 2 x 10^6 cells/ml in IMDM containing 10% FBS, 1% Pen/Strep and 10% conditioned media from BHK/MKL cells (as a source of SCF), 150 uM monothioglycerol (Sigma) and 10 ng/ml m-IL3 (PeproTech). Every 2 to 3 days cells were transferred into new media, and basophils analysed on day 7 of the differentiation (defined as live cells, GFP+, FcER1a$^+$/CD117$^-$). For *ex vivo* analysis of bone marrow basophils, flushed bone marrow cells were gated as 7AADneg, GFP$^+$, lineage (B220, CD3, CD11b, Gr-1, Ter-119) neg, FcER1a$^+$ CD117neg. Expression of IL4 and TNF was analysed by intracellular flow cytometry. In brief, cells were transferred into 96-well plates and incubated with 1 µl/ml BD GolgiPlug for 4 hours at 37° Celsius. Following live dead staining with Zombi Red (BioLegend) and subsequent staining of cell surface antigens (CD117, SiglecF and FcER1a), cells were then fixed and permeabilised using the BD Cytofix/Cytoperm™ Fixation/Permeabilization Kit, and IL4 and TNF antibodies were added (Table S1). Samples were acquired using the LSR Fortessa X-20 (BD) and data were analysed with FlowJo.

Banking and processing of human samples

Patients and healthy donors provided written informed consent in accordance with the Declaration of Helsinki for sample collection, tissue banking and use in research under the Informed study (Investigating the genetic and cellular basis of sporadic and Familial Myeloid Disorders; IRAS ID: 199833; REC reference: 16/LO/1376; PI: Prof AJ Mead). Cryopreserved bone marrow mononuclear cells isolated by density gradient centrifugation using Ficoll-Paque Premium (Sigma Aldrich) were cryopreserved in FCS with 10% DMSO (Sigma Aldrich) and thawed and processed by warming briefly at 37°C, gradually diluted into RPMI-1640 (Gibco), supplemented with 10% FCS and 0.1mg/mL DNase I (Sigma), centrifuged at 500G for 5 minutes and washed in FACS buffer (PBS + 2mM EDTA + 5% FCS).

Primary human BMSCs were isolated as previously reported (*71*). Briefly, cryopreserved mononuclear cells from bone marrow aspirates were thawed and cultured in αMEM (Gibco) supplemented with 10% FBS for 3-4 days. Subsequently non-adherent cells were removed, whereas stromal cells were selected by their adherence to plasticware.

For analysis of galectin-1 mRNA expression in platelets, published platelet RNA sequencing data was analyzed (*38*). Eligibility criteria included age ≥18 years and Stanford MPN clinic diagnosis of essential thrombocythemia, polycythemia vera or myelofibrosis (defined using the consensus criteria at the time of this study). For healthy controls, blood was collected from twenty-one asymptomatic adult donors selected at random from the Stanford Blood Center. All donors provided written consent for genetic research. For both MPN patients and healthy controls, blood was collected into acid citrate-dextrose (ACD, 3.2%) sterile yellow-top tubes (Becton, Dickinson and Co.) and platelets were isolated by established (*72-75*) purification protocols. Blood was processed within 4 h of collection for all samples. The time from whole blood collection to platelet isolation was similar between healthy donors and MPN patients.

Fibroblast to myofibroblast transition assay using human BMSC

BMSCs were seeded into collagen-treated 348 wells imaging plates (Corning cat # 356667) at 5000 cells/well and cultured for 24h in αMEM (Gibco) supplemented with 0.3% FBS, 200µM Hepes (Gibco), 50µM ß-mercaptoethanol (Gibco) and 30µg/mL ascorbic acid (Sigma Aldrich). Then media was replaced, and cells were cultured for 72h in FMT media in presence or absence of 10ng/ml TGFβ (Biolegend), 4µM OTX008 (MedChemExpress), or 20µM SB431542 (MedChemExpress). At the end of the assay, cells were fixed in cold methanol, blocked with 6% FBS in PBS and then stained for 1h with the primary antibodies for aSMA (Sigma Aldrich, 1:500) and Collagen 1 IgG1 (Sigma Aldrich, 1:4000) for 1h at RT. After that, wells were washed for 3 times with PBS and incubated with the secondary antibody Alexa488 (Thermo Scientific,

1:2000) or Alexa 568 (Thermo Scientific, 1:1000) – at RT for 2h. DRAQ5 was used to stain the nuclei. Images were acquired using the IN Cell Analyzer 6000 (GE Healthcare).

For image analysis, we used a bespoke imaging analysis program to automate calculation of the mean intensity of fluorescence for collagen 1 per well. The program takes paired grey-scale images for nuclei and collagen 1, counts the number of cells (nuclei) using edge detection and calculates the mean intensity for collagen 1 staining per cell. The source code is available at https://zenodo.org/records/13349071.

***Lgals1* expression in TNF-treated human bone marrow organoids**

Human bone marrow organoids were derived from human induced pluripotent stem cells as previously described (*42*). Mature organoids (day 18-24) were treated with TNF for 18-20h with 2ng/ml, 20ng/ml and 50ng/ml TNF (Life). After treatment, organoids were collected for RNA extraction and qRT-PCR.

Fibrosis assay with human bone marrow organoids

Human bone marrow organoids were derived from human induced pluripotent stem cells (hiPSCs) as previously described (*42*). At day 18 of the differentiation protocol, organoids were cultured for 72h with 10ng/ml TGFβ (Biolegend) in presence of 30μg/ml ascorbic acid (Sigma Aldrich), followed by 72h in the presence or absence of 10 ng/ml TGFβ, 4μM or 8μM OTX008 (MedChemExpress), or 20μM SB431542 (MedChemExpress). After treatment, organoids were collected for either fixation and imaging, or digested with 5mg/ml Collagenase II in HBSS (Sigma Aldrich) for 20min at 37°C with gentle agitation to perform RNA extraction and qRT-PCR.

For imaging, organoids were fixed in 4% PFA for 30 minutes with gentle agitation before a series of PBS washes. Washed samples were blocked in 2% goat serum, 1% BSA, Triton X100 (Sigma Aldrich), 250μL Tween-20 (Sigma Aldrich), and 500 μL sodium deoxycholate (w/v) (Sigma Aldrich) in PBS before

labelling in blocking buffer with the same antibodies used in the FMT assay. Labelled samples were then embedded in low molecular weight agarose and subject to a serial dehydration and ethyl cinnamate clearance before imaging on a Zeiss LSM 880 AiryScan confocal (*42*). For image analysis, images were processed using ImageJ/Fiji. Z stacks were subject to a maximum intensity projection before denoising and background subtraction (rolling ball). Regions of interest were drawn around organoids, and the fluorescence intensity calculated per organoid.

RNA extraction and qRT-PCR

Total RNA was isolated using the Qiagen Mini RNA isolation kit (Qiagen) and cDNA was prepared using EvoScript Universal cDNA Master (Roche) according to manufacturers' instructions. Quantitative real time PCR (qRT-PCR) was performed on a StepOne plus machine (Applied Biosystem) using the 2-ΔCt analysis method. Details of the TaqMan gene expression assays (Thermofisher Scientific) used are available in Supplemental Table 1.

Continuous Indexing of Fibrosis (CIF) scores assessment

A Learning to Rank (LTR) strategy known as RankNet (*76*)is used to assess the severity of fibrosis within and between myelofibrosis grades (MFs). The RankNet model predicts the order in which features are ranked according to their severity. A Convolutional Neural Network (CNN) is used as a feature extractor for a model to learn to rank because of its high performance in many applications, especially in medical image analysis (*77*). Therefore, the Ranking-CNN model was developed by combining RankNet with a CNN (*37*). The trained model then outputs the score. This was used as a reference for fibrosis severity which is called Continuous Indexing of Fibrosis (CIF) scores (*37*). CIF scores approach 1 when the sample is more fibrotic. To visualize the spectrum of the fibrosis within the sample, a map of fibrosis severity is generated using CIF scores.

Galectin-1 quantification

To quantify galectin-1 expression, we identified pixels with positive staining in the tissue. We applied stain deconvolution (*78*) to computationally separate the galectin-1 stain channel from the DAPI stain channel and employed stain normalization (*79*) to address staining variability across different sample batches. To identify galectin-1-positive pixels, we obtained the galectin1 pixel intensity distribution from all tissue samples and used the Otsu method (*80*) to determine an appropriate cut-off. A heatmap showing the level and variation of galectin1 expression within the same tissue is generated by calculating the ratio of galectin1-positive pixels in multiple small tissue areas (256-by-256 microns).

Analysis of *LGALS1* in human *de novo* AML and leukemic transformation of MPN

TCGA AML patient survival data and gene expression values were retrieved as previously described (*81*). In total, 132 patients with survival and gene expression data were available and included for survival and gene set enrichment analysis (GSEA) (*82*). To interrogate *LGALS1* expression in patients with blast phase MPN, we explored published RNA-sequencing data of CD34+ HSPCs and total mononuclear cells from patients with accelerated/blast phase MPN (AP/BP-MPN, n = 10) and healthy donors (n = 5) (*46*).

For survival analysis, we stratified the patients into *LGALS1*-high and *LGALS1*-low based on the median gene expression value of *LGALS1* across the cohort. Cox proportional hazards regression model was fitted using the coxph function from the R package survival to estimate the hazard ratio of *LGALS1*-high patients by using *LGALS1*-low patients as the reference group. The Kaplan-Meier (KM) curve was plotted using the ggsurvplot function from the survminer R package.

For GSEA, we first identified differentially expressed genes between the *LGALS1*-high and *LGALS1*-low patient groups. To this end, we included genes that were expressed in at least 10% of patients in either group. A gene was considered to be expressed when its FPKM (Fragments Per Kilobase of transcript

per Million mapped reads) value was at least 1 and above. The gene expression values were subsequently offset by +1 and log2-transformed. Then, the expression values of each gene were compared between the two patient groups using t-test and the log2(fold change) was computed for *LGALS1*-high relative to *LGALS1*-low patient group using the mean expression value of each patient group. Next, a score was generated for each gene by computing -log10(P value) and then multiplying by -1 if the log2fc < 0. This score was used to rank the genes from the most-regulated to most-downregulated in *LGALS1*-high relative to *LGALS1*-low patient group, and subsequently used as input for GSEA software. Our ranked gene list was assessed for enrichment of the HALLMARK gene set using RunGSEAPreranked option. Gene sets with false discovery rate (FDR) < 0.25 were considered to be significantly enriched among our ranked gene list.

SUPPLEMENTARY FIGURES

Figure S1

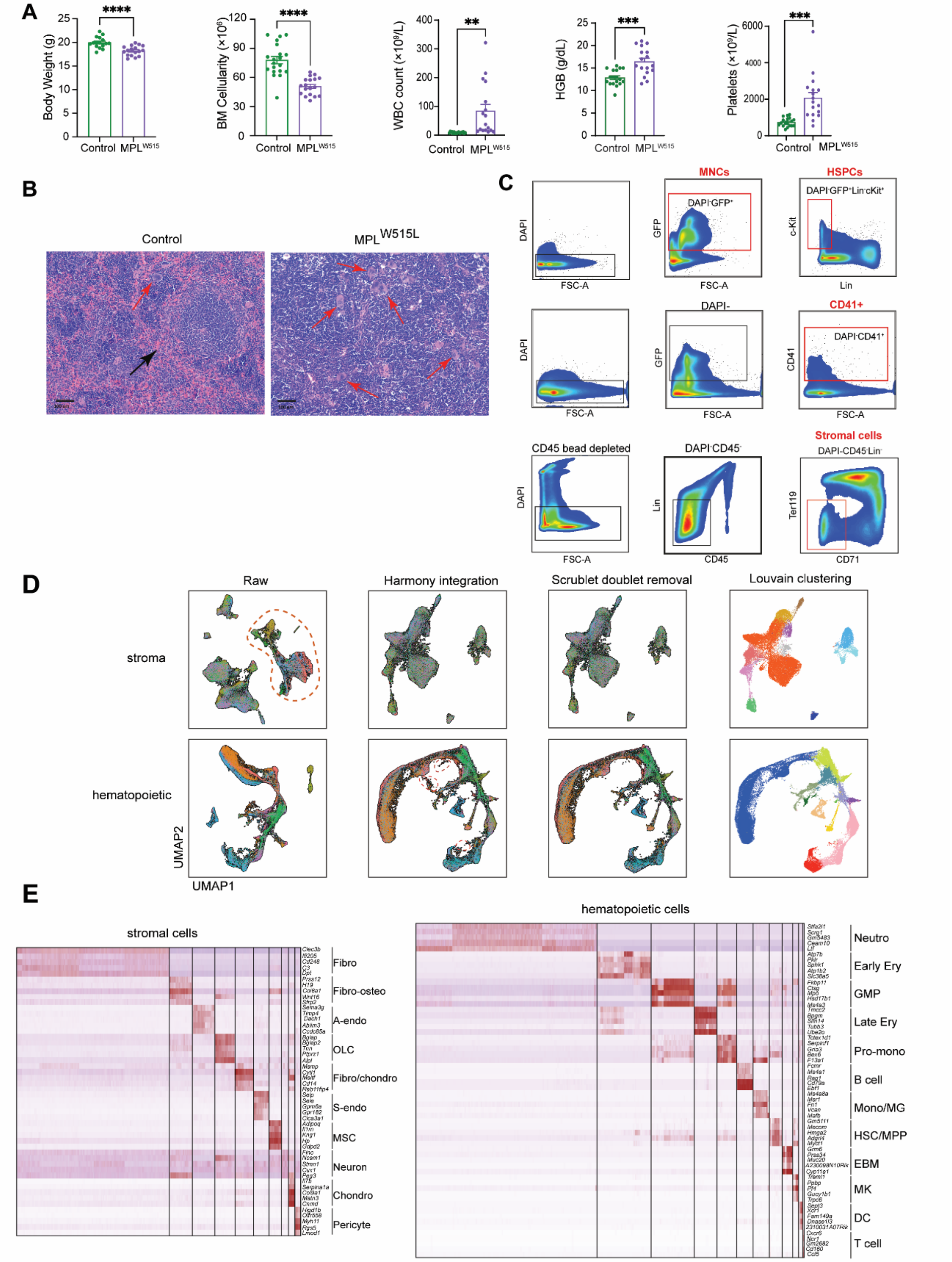

S1. A comprehensive map of myelofibrotic bone marrow, relating to Figure 1. (**A**) Bar charts showing the body weight of control (n=16) and MPL^{W515L} (n=17) mice, total bone marrow cellularity of 2 femurs and 2 tibias in control (n=20) and MPL^{W515L} mice (n=18), white blood cell count, haemoglobin and platelet counts in control (n=18) and MPL^{W515L} mice (n=17). **p < 0.01, ***p < 0.001, ****p < 0.0001 for unpaired t test with Welch's correction. (**B**) Representative images of haematoxylin & eosin stained spleen from control mice (n=16) and MPL^{W515L} mice. (n=19). Scale bar, 100μm. Red arrows indicate megakaryocytes, and black arrow indicates red pulp. (**C**) Gating strategy for sorting total mononuclear cells, hematopoietic stem/progenitor cells (HSPCs), CD41+ cells and stromal cells for single cell RNA-sequencing. (**D**) Integration of scRNAseq datasets from 3 experiments (Harmony) and removal of doublets (using Scrublet package), prior to louvain clustering. (**E**) Heatmaps showing 5 top differentially expressed genes in each annotated cell type for stromal (lea) and hematopoietic cells (right). Abbreviations: BM, bone marrow; MNC, mononuclear cells; Fibro-chondro, fibroblast-chondrocytes; Chondro, chondrocytes; OLC, osteolineage cells; Fibro-osteo, fibroblast-osteoblasts; Fibro, Fibroblasts; MSC, mesenchymal stromal cells; A-endo, arterial endothelial cells; S-endo, sinusoidal endothelial cells; Neutro, neutrophils; GMP, granulocyte-monocyte progenitors; Pro-mono, monocyte progenitors; Mono/MG, monocyte/macrophages; HSC/MPP, hematopoietic stem and multipotent progenitor cells; MK, megakaryocytes; EBM, eosinophil, basophil, mast cells; DC, dendritic cells; B, B cells; T, T cells; Ery, erythrocytes.

Figure S2

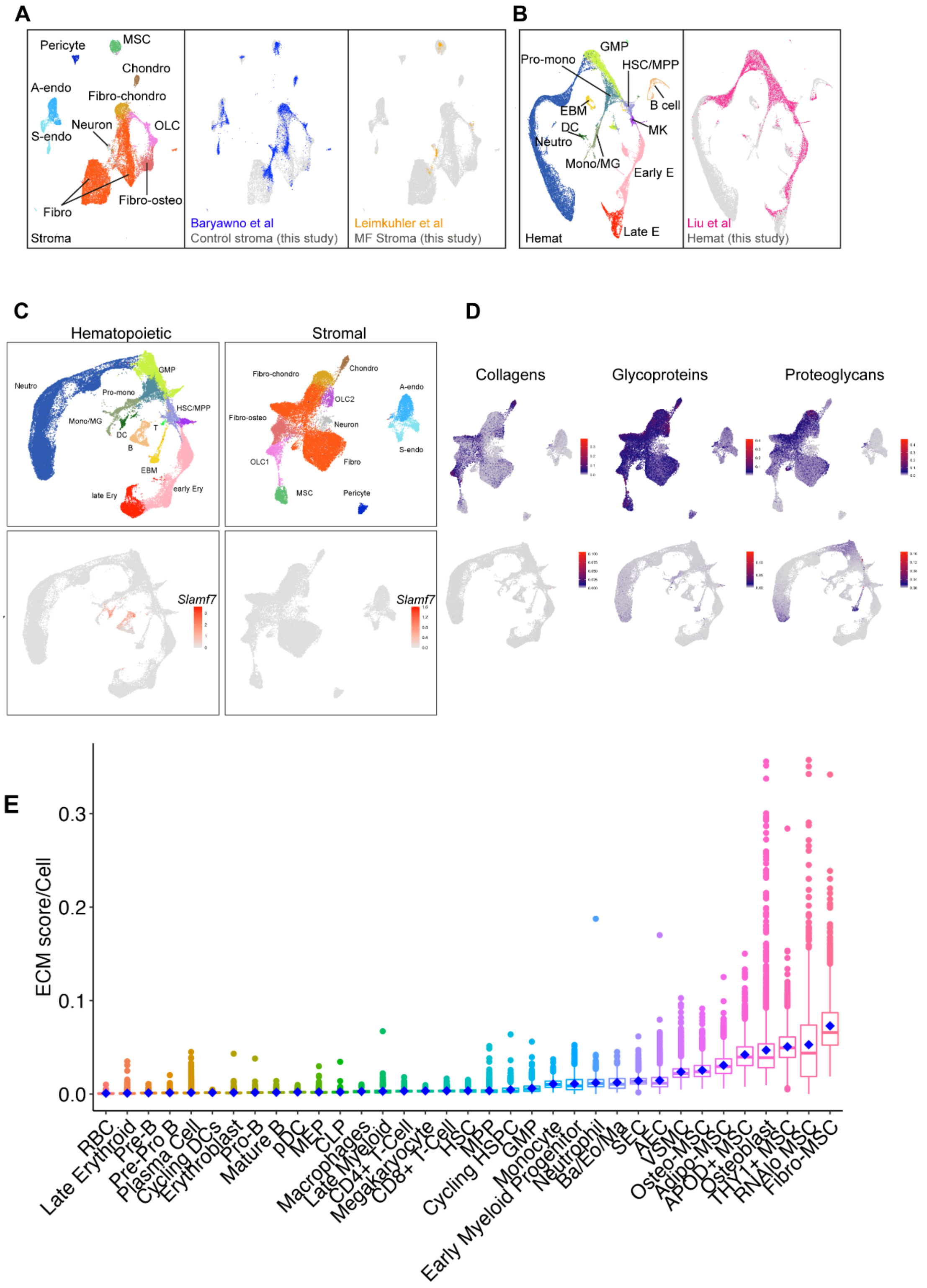

Figure S2. Comparison of current dataset to previously published annotations of normal and myelofibrotic bone marrow, and expression of extracellular matrix (ECM) components, relating to

Figures 1 and 2. (**A & B**) Comparison of the stromal (**A**) and haematopoietic (**B**) cells captured to previously published studies by projecting healthy (Baryawano *et al*, blue) and myelofibrotic mouse bone marrow (Leimkuhler *et al*, orange containing stromal cells and Liu *et al,* pink containing haematopoietic cells) onto a reference Uniform Manifold Approximation and Projection (UMAP) plot generated using the cells captured by our study (grey). Abbreviation: Hemat, haematopoietic cells. (**C**) UMAPs showing expression of monocyte marker Slamf7 in stromal cell and haematopoietic cell subsets. (D) UMAPs showing expression of extracellular matrix (ECM) factors in stromal and hematopoietic cell subsets, broken down into collagens, glycoproteins and proteoglycans. (**E**) Gene expression of ECM factors in hematopoietic and stromal cell subsets from a published atlas of healthy human bone marrow (Bandyopadhay et al, Cell 2024).

Figure S3

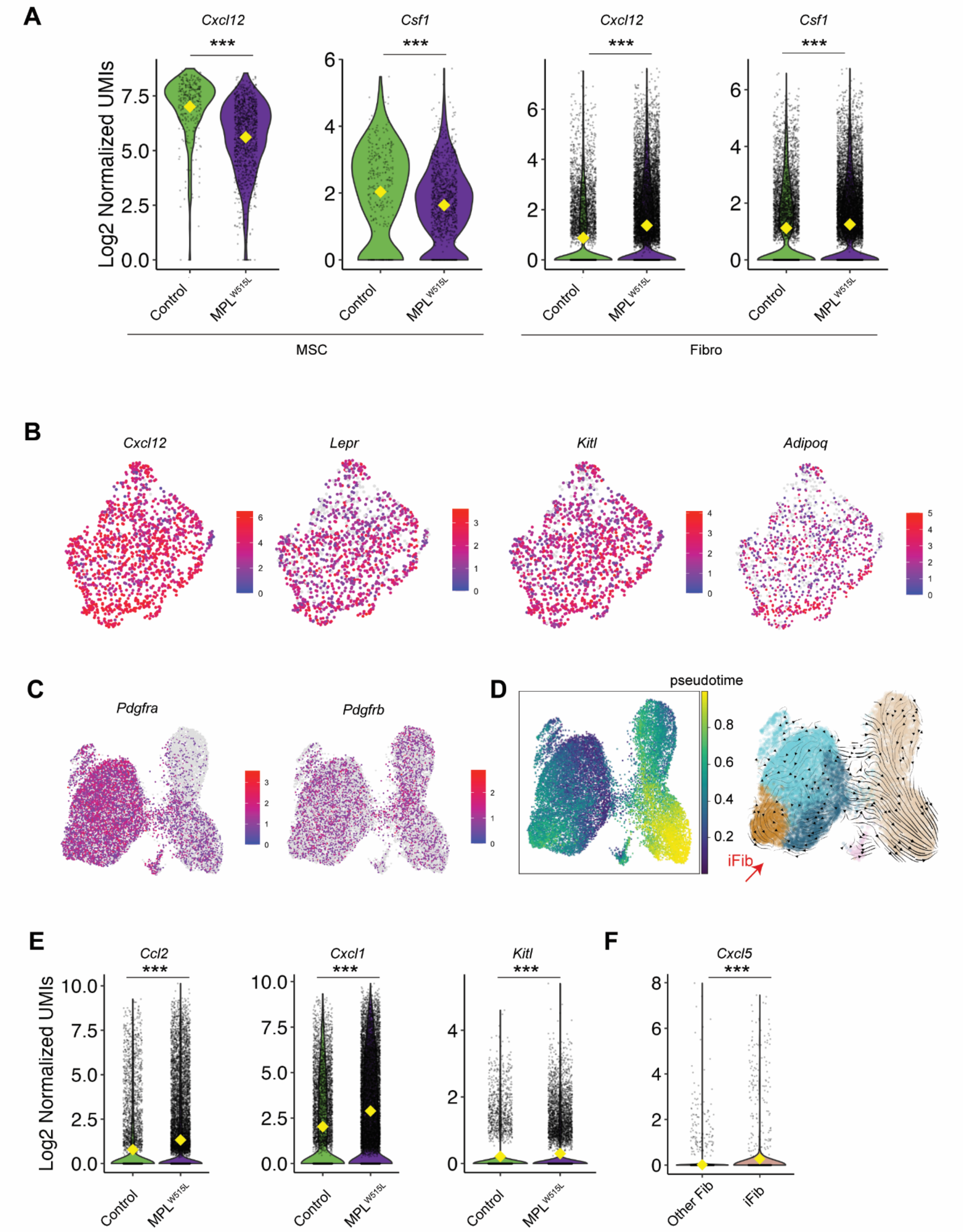
A
Cxcl12
Csf1
Cxcl12
Csf1
***
Log2 Normalized UMIs
Control
MPL W515L
MSC
Fibro
B
Cxcl12
Lepr
Kitl
Adipoq
C
Pdgfra
Pdgfrb
D
pseudotime
iFib
E
Ccl2
Cxcl1
Kitl
F
Cxcl5
Other Fib
iFib

Figure S3. Trans-differentiation of mesenchymal stromal cells and expansion of inflammatory fibroblasts in myelofibrosis, relating to Figure 3. (**A**) Violin plots showing expression of *Cxcl12* and *Csf1* in mesenchymal stromal cells (MSC) (lea) and fibroblasts (Fibro) (right). Yellow diamond indicates mean value. ***p < 0.001 for Wilcoxon test. (**B**) Uniform Manifold Approximation and Projections (UMAPs) showing expression of canonical MSC marker genes (*Lepr, Adipoq*) and hematopoietic support factors (*Cxcl12 and Kitl*) in MSCs extracted from the main stromal cell dataset.

(**C**) Expression of canonical fibroblast marker *Pdgfra and Pdgfrb* on UMAP. (**D**) Pseudotime analysis of fibroblasts using scTour showing that inflammatory fibroblasts (iFib) arise via a separate trajectory from Fib1 cluster (enriched in control mice). The blue cluster indicates the Fib1 cluster.

(**E**) Violin plots showing expression of selected chemokine genes (*Ccl2, Cxcl1 and Kitl*) in fibroblasts in control and MPL^{W515L} mice. Yellow diamond indicates mean value. ***p < 0.001 for Wilcoxon test. (**F**) Violin plot showing expression of *Cxcl5* in iFib vs. all other fibroblasts. Yellow diamond indicates mean value. ***p < 0.001 for Wilcoxon test.

Figure S4

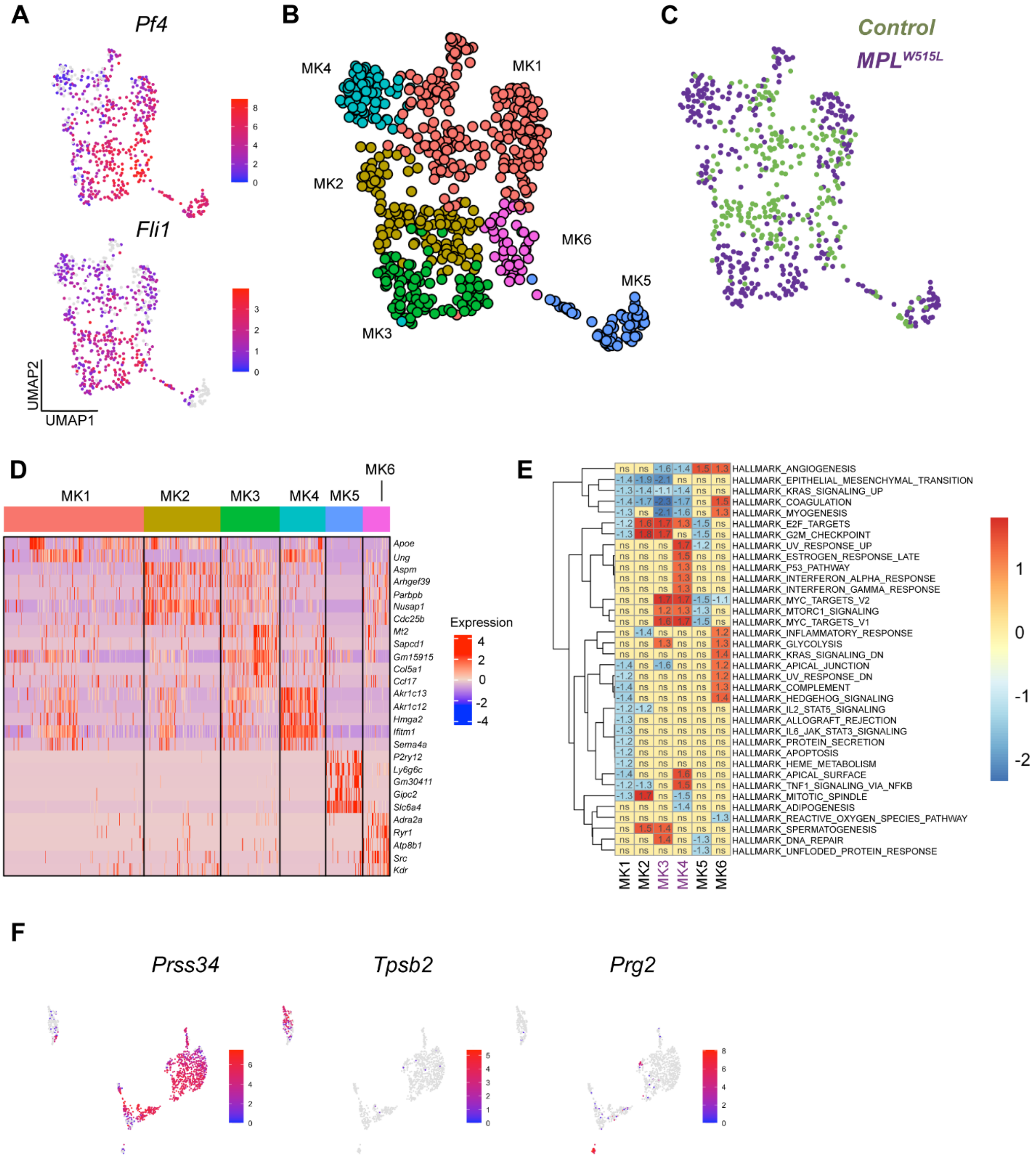

Figure S4. Myelofibrosis megakaryocytes, mast cells and basophils show inflammatory transcriptional programs in myelofibrosis, relating to Figure 4. (**A**) Uniform manifold approximation and projection (UMAP) plots showing extracted megakaryocytes from the main dataset, confirming high

expression of canonical marker genes *Pf4* and *Fli1*. (**B**) Unsupervised clustering of extracted megakaryocytes identified 6 distinct subtypes. (**C**) Identification of megakaryocytes captured from MPL^{W515L} (purple) and control (green) mice. (**D**) Top 5 differentially expressed genes in each megakaryocyte subcluster. (**E**) Heatmap showing gene set enrichment analysis for HALLMARK gene sets in each megakaryocyte subcluster. (**F**) Canonical marker genes for basophils (*Prss34*), mast cells (*Tpsb2*) and eosinophils (*Prg2)* respectively shown on EBM cell UMAP.

Figure S5

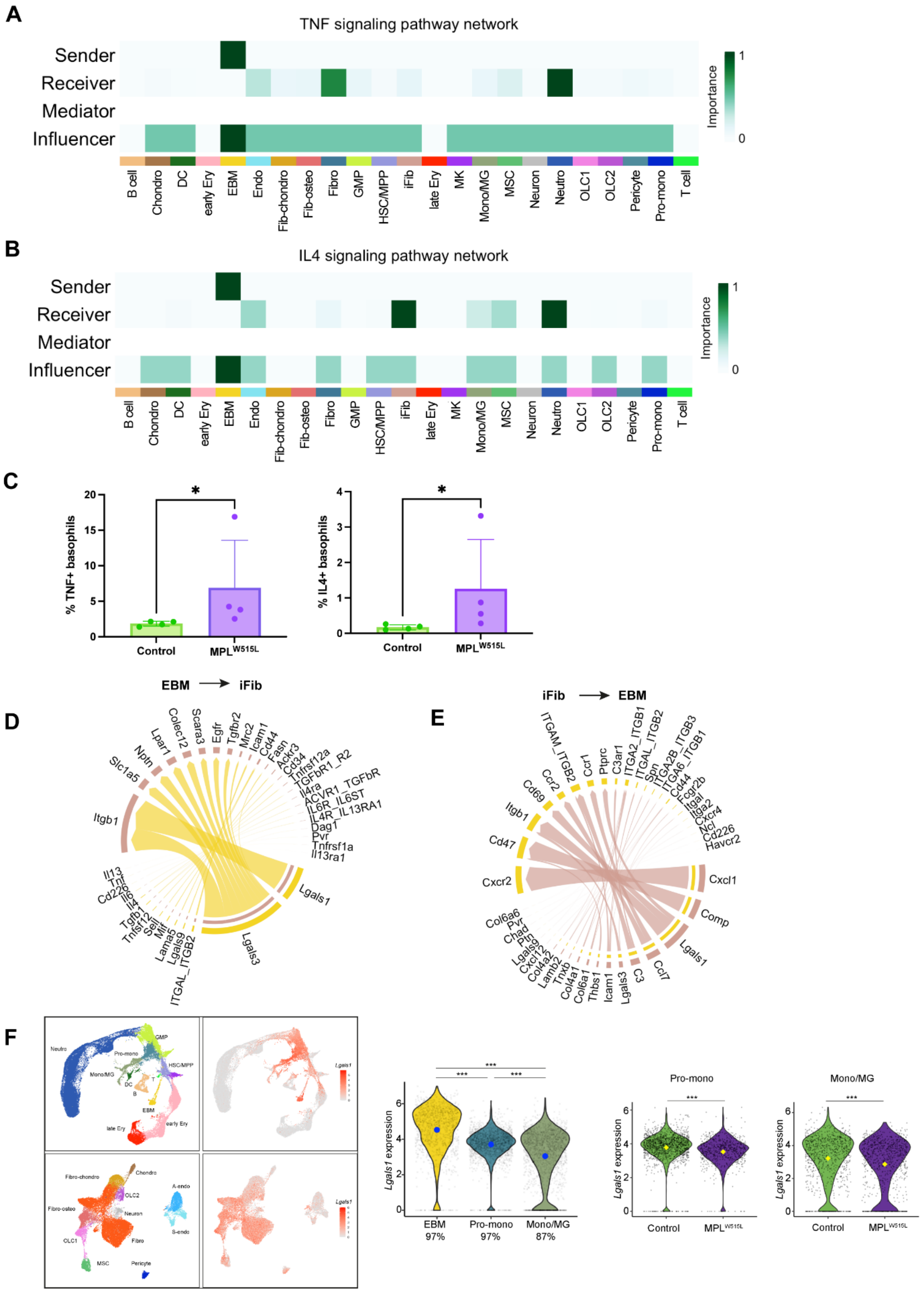
A
TNF signaling pathway network
Sender
Receiver
Mediator
Influencer
Importance
1
0
B cell
Chondro
DC
early Ery
EBM
Endo
Fib-chondro
Fib-osteo
Fibro
GMP
HSC/MPP
iFib
late Ery
MK
Mono/MG
MSC
Neuron
Neutro
OLC1
OLC2
Pericyte
Pro-mono
T cell
B
IL4 signaling pathway network
C
% TNF+ basophils
% IL4+ basophils
Control
MPLW515L
D
EBM → iFib
Itgb1
Slc1a5
Nptn
Lpar1
Colec12
Scara3
Egfr
Tgfbr2
Mrc2
Icam1
Cd44
Fasn
Ackr3
Cd34
Tnfrsf12a
TGFbR1_R2
Il4ra
ACVR1_TGFbR
IL6R_IL6ST
IL4R_IL13RA1
Dag1
Pvr
Tnfrsf1a
Il13ra1
Lgals1
Lgals3
ITGAL_ITGB2
Lgals9
Lama5
Mif
Sell
Tnfsf12
Tgfb1
Il4
Il6
Cd226
Tnf
Il13
E
iFib → EBM
ITGAM_ITGB2
Ccr2
Cd69
Itgb1
Cd47
Cxcr2
Ccr1
Ptprc
C3ar1
ITGA2_ITGB1
ITGAL_ITGB2
Spn
ITGA2B_ITGB3
ITGA6_ITGB1
Cd44
Fcgr2b
Itgal
Itga2
Cxcr4
Ncl
Cd226
Havcr2
Cxcl1
Comp
Lgals1
Ccl7
C3
Lgals3
Icam1
Thbs1
Col6a1
Col4a1
Tnxb
Lamb2
Col4a2
Cxcl12
Lgals9
Ptn
Chad
Pvr
Col6a6
F
Neutro
Pro-mono
GMP
HSC/MPP
Mono/MG
DC
B
T
EBM
late Ery
early Ery
Fibro-chondro
Chondro
OLC2
A-endo
Fibro-osteo
Neuron
S-endo
OLC1
Fibro
MSC
Pericyte
Lgals1
Lgals1 expression
EBM
97%
Pro-mono
97%
Mono/MG
87%
Pro-mono
Mono/MG
Control
MPLW515L

Figure S5. Up-regulated receptor-ligand interactions in myelofibrotic bone marrow, relating to Figure 5. (**A & B)** Interaction network of **(A)** TNF and (**B**) IL4 signalling pathway indicating EBM cluster is the key ligand resource (sender) for both pathways. (**C**) Percentage of TNF-positive (left) and IL4-positive (right) basophils measured by intracellular flow cytometry of cells differentiated *in vitro* from control and MPL^{W515L} stem/progenitor cells (n=4 each group). (**D** & **E**) Circus plot depicting upregulated interaction pairs in MPL^{W515L} vs control mice between EBM cluster and iFib cluster highlighting the enrichment of *Lgals1* interactions. **(F)** Left - UMAPs showing expression of *Lgals1* in hematopoietic and stromal cell subtypes; centre – violin plots showing expression of *Lgals1* in eosinophil-basophil-mast (EBM) cell, pro-monocyte (pro-mono) and monocyte/macrophage (mono/MG) cell clusters; right – expression of *Lgals1* in pro-mono and mono/MG cells in control vs. MPL^{W515L} mice. *** $p < 0.001$.

Figure S6

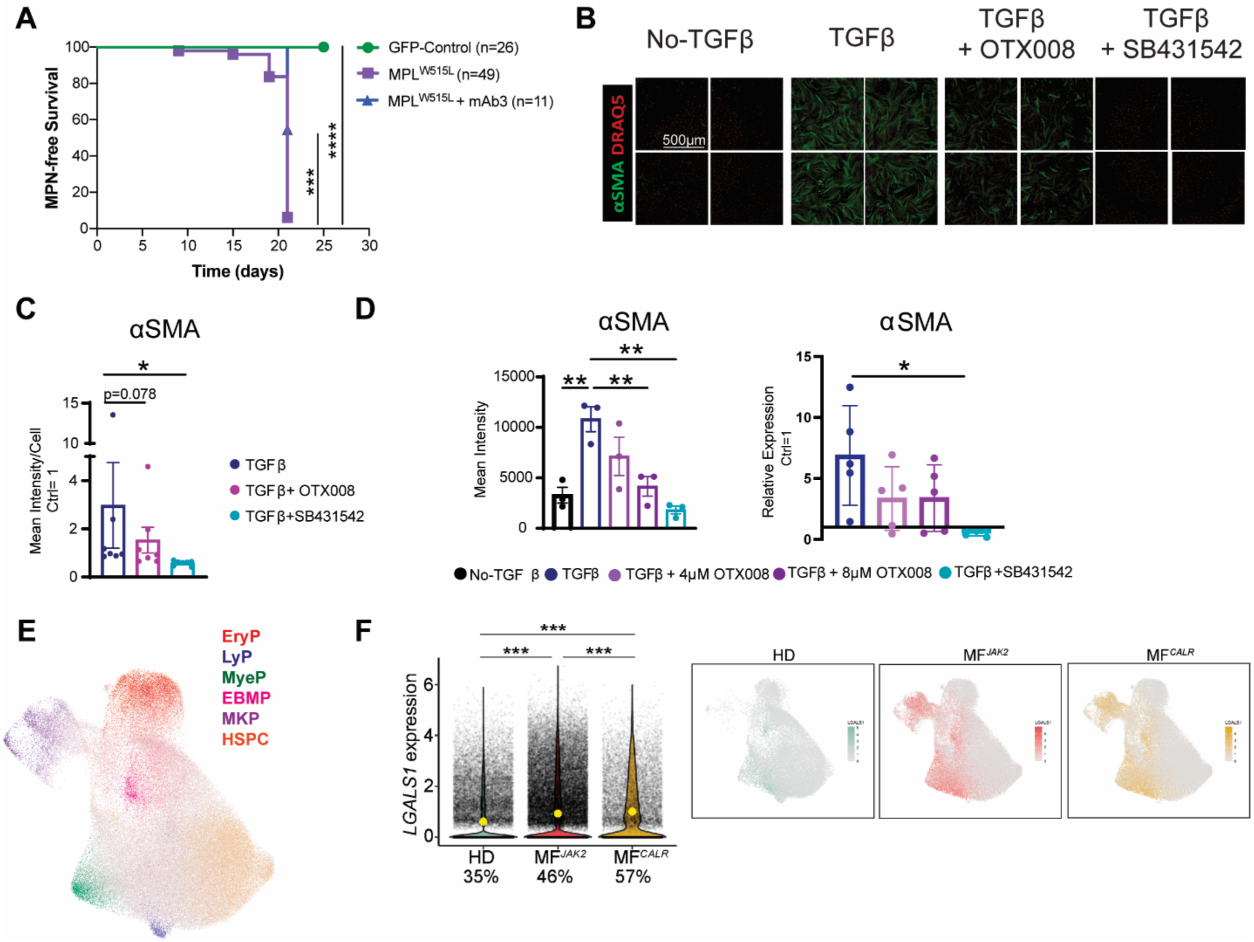

Figure S6. Inhibition of galectin 1 signalling reduces myelofibrosis phenotype *in vitro* and *in vivo*, relating to Figure 7. (A) Kaplan–Meier curve showing MPN-free survival (defined by blood parameters and spleen size) for control mice (n=26), MPL^{W515L} mice (n=49) and anti-Gal1 mAb3 treated MPL^{W515L} mice (n=11). *** p < 0.001, **** p < 0.0001 for Gehan-Breslow-Wilcoxon test. **(B)** TGFβ-induced fibroblast to myofibroblast transition assay using human bone marrow stromal cells treated with TGFβ alone with the galectin 1 inhibitor OTX008 or the TGFβ inhibitor SB431542. Representative images shown (left) from high-throughput 384-well plate assay. (C) Chart shows Mean Fluorescence Intensity per cell for αSMA normalized to the no-TGFβ control ± SEM (n=7). *p < 0.05 for wilcoxon matched pairs signed rank test. (D) αSMA protein quantification by immunofluorescence staining intensity and RT-PCR for gene expression of human bone marrow organoids treated with TGFβ to induce organoid fibrosis + OTX008 or

SB431542. n=5-8 organoids from 3 independent experiments. *p < 0.05, ** p < 0.01 for one-way ANOVA. (E) Identification of eosinophil, basophil and mast cell progenitors (EBMP) within a published dataset of >120,000 CD34+ Lineage negative hematopoietic stem/progenitor cells from patients with myelofibrosis and age-matched healthy donors. **(**F**) Left-**Expression of *LGALS1* in HSPCs from patients with myelofibrosis driven by JAK2V617F and mutant CALR. *** p < 0.001. Right- Expression of *Lgals1* in hematopoietic stem/progenitor cells (HSPCS, left, 200 cell 'minibulks', n = 5 healthy controls; n = 10 accelerated/blast phase MPN, AP/BP-MPN).

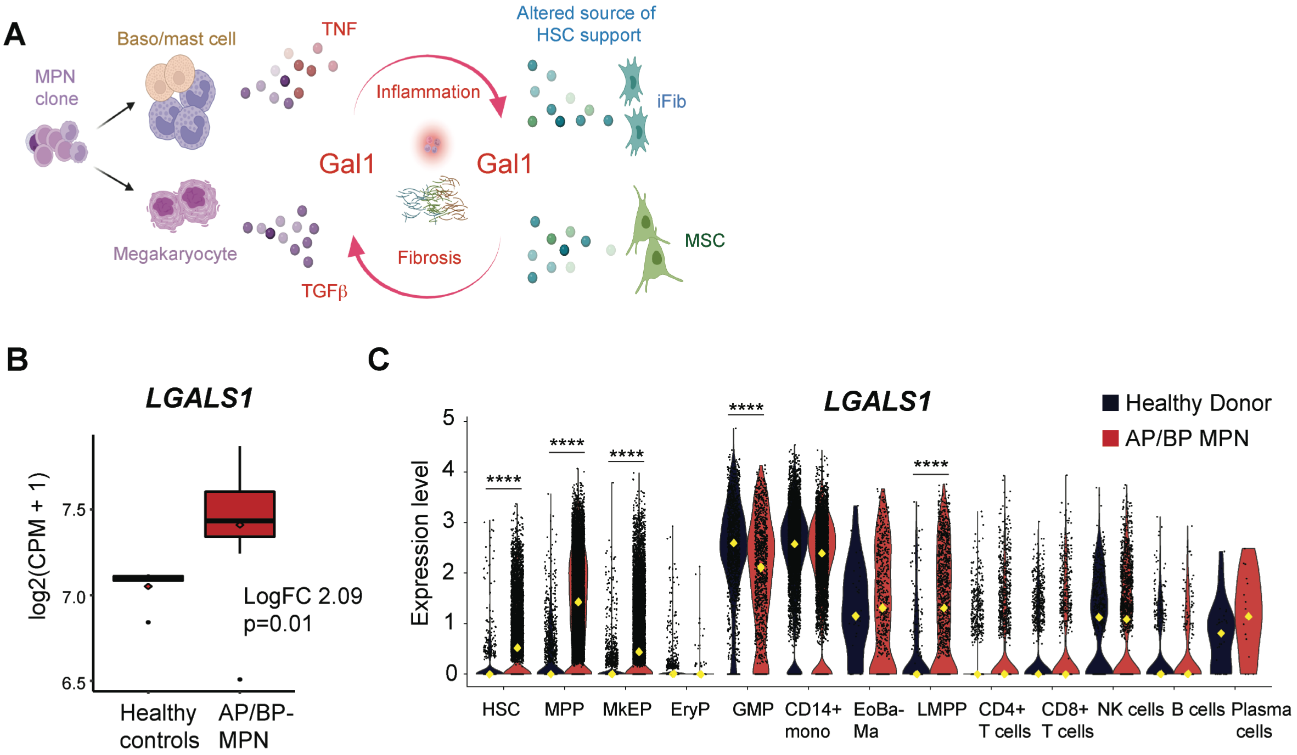

	HSC	MPP	MkEP	EryP	GMP	CD14 Mono	EoBaMa	LMPP	CD4+T cells	CD8+T cells	NK cells	B cells	Plasma cells
HD	136/492 (27.6%)	442/1006 (43.9%)	388/1172 (33.1%)	224/821 (27.3%)	1336/1400 (95.4%)	4560/5077 (89.8%)	26/32 (81.2%)	77/213 (36.2%)	301/1245 (24.2%)	352/1230 (28.6%)	343/562 (61%)	128/428 (29.9%)	25/35 (71.4%)
AP/BP MPN	4381/7211 (60.8%)	12639/15120 (83.6%)	5477/8986 (61%)	23/158 (14.6%)	977/1110 (88%)	4879/5728 (85.2%)	427/597 (71.5%)	786/1103 (71.3%)	493/1446 (34.1%)	286/858 (33.3%)	857/1526 (56.2%)	73/247 (29.6%)	14/17 (82.4%)

Figure S7. Galectin-1 fuels progression of MPNs to myelofibrosis and is highly expressed in stem/progenitor cells in accelerated/blast phase MPN. **(A)** Schematic illustrating the interactions between basophils, mast cells and megakaryocytes derived from the MPN clone interacting with BMSC subsets, fueling inflammation and fibrosis via galectin-1 induction. Created with Biorender.com. Abbreviations: Ctrl, control; TGFβ, transforming growth factor β; Col1, collagen 1; aSMA, alpha smooth muscle actin; anti-Gal-1, monoclonal anti-Galectin-1 neutralizing antibody; HCT, hematocrit; IgG, isotype

IgG control antibody; H&E, hematoxylin and eosin; g, grams. (**B**) Expression of *LGALS1* in HSPCs from patients with myelofibrosis driven by JAK2V617F and mutant CALR. *** $p < 0.001$. (**C**) Expression of *LGALS1* in HSPC subsets and mature blood cell lineages in single cell RNA sequencing data from healthy donors (HD, n = 5) and patients with AP/BP-MPN, n = 10). Violin plots show RNA expression, diamond indicates mean value and **** $p < 0.0001$. Table below indicates number and % of *LGALS1*-expressing cells for each group out of all cells analysed.

Data files

Data file S1: Antibody and chemical list

Data file S2: Differentially expressed genes in stromal and hematopoietic cell clusters

Data file S3: Details of patient samples used

Data file S4: Genesets used for chemokine, niche supporting factor scores and to identify eosinophil, basophil and mast cell progenitors in human CD34+ lineage negative hematopoietic stem & progenitor cells

Data file S5: Details of samples submitted to GEO

Data file S6: Raw data for experiments with n<20
